# Endosomal traffic and glutamate synapse activity are increased in VPS35 D620N mutant knock-in mouse neurons, and resistant to LRRK2 kinase inhibition

**DOI:** 10.1186/s13041-021-00848-w

**Published:** 2021-09-16

**Authors:** Chelsie A. Kadgien, Anusha Kamesh, Austen J. Milnerwood

**Affiliations:** 1 Graduate Program in Neuroscience and Centre for Applied Neurogenetics, Djavad Mowafaghian Centre for Brain Health, University of British Columbia, Vancouver, Canada; 2 Montreal Neurological Institute-Hospital, McGill University, Montreal, Canada

**Keywords:** Parkinson's disease, Alpha-amino-3-hydroxy-5-methyl-4-isoxazolepropionic acid (AMPA), NMDA, Dopamine Receptor, Glutamate, Synapses, Synaptic transmission, Knock-in mouse, Retromer, Protein traffic

## Abstract

Vacuolar protein sorting 35 (VPS35) regulates neurotransmitter receptor recycling from endosomes. A missense mutation (D620N) in VPS35 leads to autosomal-dominant, late-onset Parkinson’s disease. Here, we study the basic neurobiology of VPS35 and Parkinson’s disease mutation effects in the D620N knock-in mouse and the effect of leucine-rich repeat kinase 2 (LRRK2) inhibition on synaptic phenotypes. The study was conducted using a VPS35 D620N knock-in mouse that expresses VPS35 at endogenous levels. Protein levels, phosphorylation states, and binding ratios in brain lysates from knock-in mice and wild-type littermates were assayed by co-immunoprecipitation and western blot. Dendritic protein co-localization, AMPA receptor surface expression, synapse density, and glutamatergic synapse activity in primary cortical cultures from knock-in and wild-type littermates were assayed using immunocytochemistry and whole-cell patch clamp electrophysiology. In brain tissue, we confirm VPS35 forms complexes with LRRK2 and AMPA-type glutamate receptor GluA1 subunits, in addition to NMDA-type glutamate receptor GluN1 subunits and D2-type dopamine receptors. Receptor and LRRK2 binding was unaltered in D620N knock-in mice, but we confirm the mutation results in reduced binding of VPS35 with WASH complex member FAM21, and increases phosphorylation of the LRRK2 kinase substrate Rab10, which is reversed by LRRK2 kinase inhibition in vivo. In cultured cortical neurons from knock-in mice, pRab10 is also increased, and reversed by LRRK2 inhibition. The mutation also results in increased endosomal recycling protein cluster density (VPS35-FAM21 co-clusters and Rab11 clusters), glutamate transmission, and GluA1 surface expression. LRRK2 kinase inhibition, which reversed Rab10 hyper-phosphorylation, did not rescue elevated glutamate release or surface GluA1 expression in knock-in neurons, but did alter AMPAR traffic in wild-type cells. The results improve our understanding of the cell biology of VPS35, and the consequences of the D620N mutation in developing neuronal networks. Together the data support a chronic synaptopathy model for latent neurodegeneration, providing phenotypes and candidate pathophysiological stresses that may drive eventual transition to late-stage parkinsonism in VPS35 PD. The study demonstrates the VPS35 mutation has effects that are independent of ongoing LRRK2 kinase activity, and that LRRK2 kinase inhibition alters basal physiology of glutamate synapses in vitro.

**Supplementary Information:**

The online version contains supplementary material available at 10.1186/s13041-021-00848-w.

## Introduction

Vacuolar protein sorting 35 (VPS35) is a core component of the retromer complex, which recycles transmembrane cargo from endosomes to the *trans-*Golgi network (TGN) or surface plasma membranes, circumventing lysosomal degradation (reviewed in: 1–4). A missense mutation in VPS35 (D620N) causes late-onset autosomal Parkinson’s disease (PD) that is clinically indistinguishable from idiopathic PD [5–7]. PD is classically thought of as a motor disorder caused by degeneration of dopamine neurons in the *substantia nigra*; however, disease progression continues after functional loss of nigral projections to the striatum [8]. PD is accompanied by non-motor symptoms that can precede motor onset by decades, and that are not responsive to dopamine replacement therapies (reviewed in: 9,10). The disease is also characterized by impaired cortical synaptic plasticity occurring prior to motor symptom onset and cortical neuron loss at later stages (reviewed in: 11). Such observations highlight the involvement of the glutamatergic system early in the disease process, and throughout its progression. VPS35 D620N knock-in (VKI) mice eventually develop nigral pathology [12,13] validating their relevance as models of PD, and here we investigate whether the VPS35 mutation impacts glutamatergic function in cortical neurons. We identify early alterations that might be used as biomarkers of mutant effects and targeted to prevent transition to later disease states.

In neurons, retromer clusters are highly motile in soma, axons, dendrites, and dendritic spines [14,15]. We, and others, demonstrated by overexpression and knock-out studies that VPS35 participates in surface delivery of GluA1 subunit-containing alpha-amino-3-hydroxy-5-methyl-4-isoxazolepropionic acid (AMPA)-type glutamate receptors, supports synapse development, maturation, and activity-dependent AMPA receptor (AMPAR) delivery required for the expression of long-term potentiation (LTP; 14–22). We found that exogenous expression of D620N mutant VPS35 impairs its motility in dendrites and trafficking into spines [15]. Furthermore, we previously showed mutant VPS35 expression altered excitatory synaptic current amplitudes in mouse neurons, and AMPA receptor cluster intensities in both mouse neurons and dopamine neuron-like cells derived from human mutation carrier induced pluripotent stem cells [15]. Together, this argues retromer is important in synapse development, maintenance, and functional connectivity. However, it was previously unclear the extent to which overexpression artefacts might impact physiological retromer function, and how endogenous mutations in VPS35 might alter its role at the synapse.

VPS35 interacts with leucine-rich repeat kinase 2 (LRRK2), another PD-implicated protein, both physically and functionally [23–28]. LRRK2 is a large multi-domain protein implicated in ~ 5% of all familial Parkinson’s disease through autosomal-dominant mutations and genetic risk, the most common mutation being the G2019S substitution [29]. In neurons, changes to LRRK2 levels, and LRRK2 mutations, dysregulate synaptic vesicle (SV) recycling and release [30–34], post-synaptic receptor trafficking, and dendritic spine development [35–38]. Inoshita and colleagues [25] recently reported that LRRK2 localizes with VPS35 at *Drosophila* neuromuscular junctions where they are proposed to work together regulate the SV cycle.

PD-associated mutations in LRRK2 increase its kinase activity (reviewed in: 39), which hyperphosphorylates multiple LRRK2 substrates (reviewed in: 40). The best-characterized LRRK2 substrates are several Rab-GTPases [41,42]. Increased autophosphorylation of LRRK2 and phosphorylation of Rab10 have been observed in *post-mortem substantia nigra* from individuals with idiopathic PD [43], monocytes from humans with the D620N mutation, and tissues from VPS35 D620N knock-in mice [27]. This provides evidence that VPS35 and LRRK2 mutations converge on LRRK2 kinase activity, and that aberrant phosphorylation of Rab proteins involved in synaptic transmission may be related to the several synaptic phenotypes observed in PD models (reviewed in: 40).

Here we probe the neurobiological function of VPS35 in developing neural networks, potentially pathophysiological dysfunction, and drug responses in neurons from VKI mice. This may uncover factors that combine with age and other environmental stresses to eventually trigger transition to pathology. We examined synapse development, structure, and function, in addition to protein levels, phosphorylation state, and binding relationships in brain tissue and cultured cortical neurons. The D620N mutation reduced retromer complex association with its regulatory proteins by coIP, increased dendritic clustering of proteins involved in surface protein recycling, and augmented glutamate transmission. We assayed Rab10 phosphorylation in brain and cultured cortical neurons and the effects of LRRK2 kinase inhibition on glutamatergic transmission phenotypes. LRRK2 kinase inhibition did not rescue mutant synaptic phenotypes. In wild-type cells, LRRK2 kinase inhibition produced effects consistent with LRRK2 negatively regulating the delivery of AMPARs to developing/silent synapses.

VKI mice provide a model system in which to develop insights into the molecular and cellular effects of VPS35 mutations, and potentially the etiology PD. Dysregulation of glutamate transmission in VKI mice resembles that reported in LRRK2 G2019S knock-in mouse models of parkinsonism; however, the failure of LRRK2 kinase inhibition to reverse glutamate phenotypes in VKI mice suggests VPS35 D620N mutation effects do not result from ongoing LRRK2 kinase activity or are not rapidly reversible. Our findings extend our understanding of VPS35 neurobiology, and have important implications for interpretation of mutation effects induced by exogenous protein expression and the potential utility of LRRK2 kinase inhibitors in the treatment of non-LRRK2 PD.

## Results

### Altered protein binding relationships and LRRK2 kinase activity in VKI brain

**Fig. 1:**
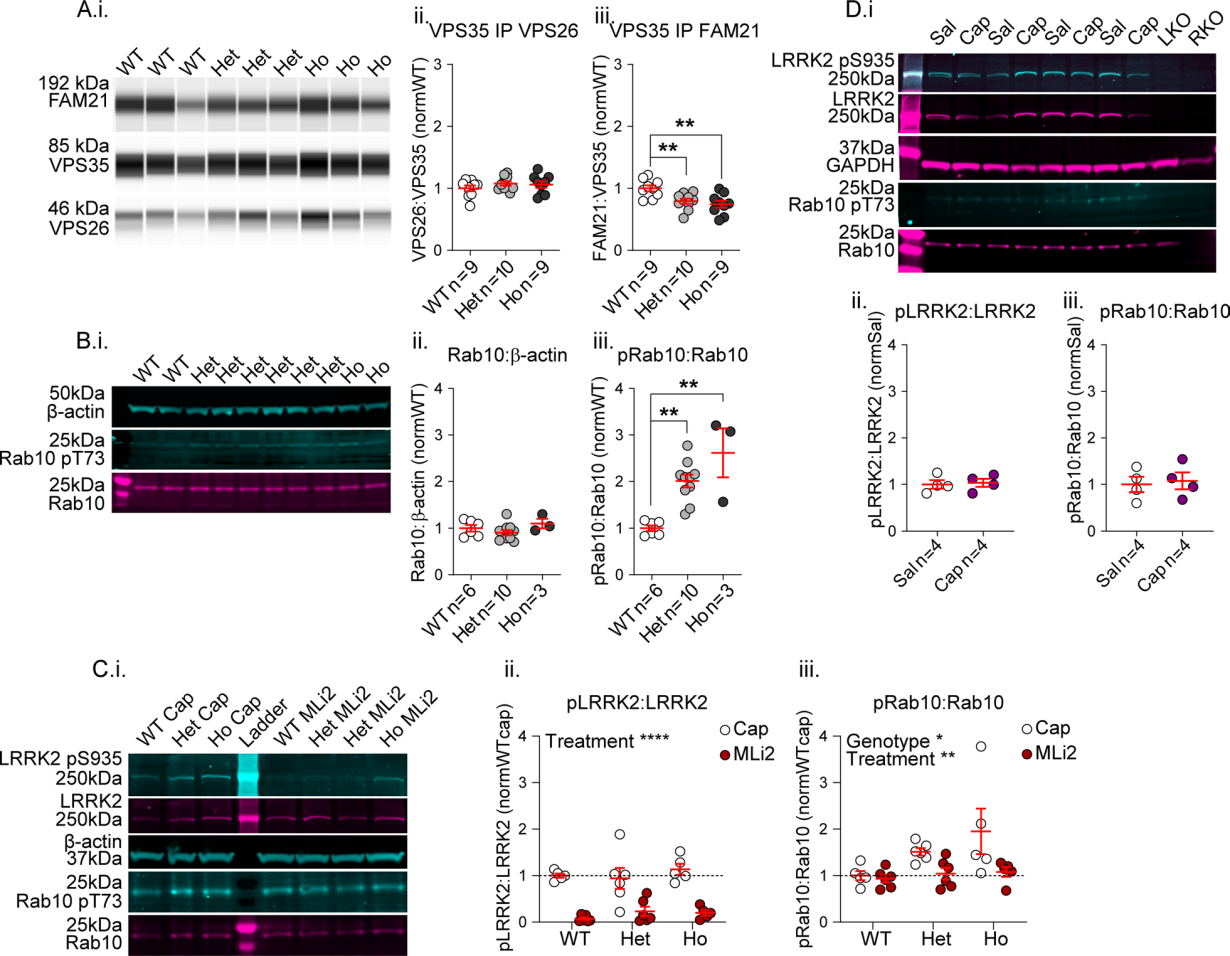
Altered FAM21 binding and LRRK2 kinase inhibitor-reversible Rab-GTPase phosphorylation in VKI mouse brain. **A** Co-immunoprecipitations of VPS26 and FAM21 with VPS35 from VKI whole-brain lysate run on a WES capillary-based western blot system (i) uncovered no effect on VPS26 pulled by VPS35 (ii, 1-way ANOVA *p* = 0.44) but a significant reduction in FAM21 pulled by mutant VPS35 (iii, 1-way ANOVA *p* < 0.003; Uncorrected Fisher’s LSD Het **p < 0.01; Ho **p < 0.01). **B** Western blot of Rab10, Rab10 pT73, and β-actin in VKI whole brain lysate (i) revealed no significant genotype effect on Rab10 levels (ii, Kruskal–Wallis *p* = 0.16), but a significant increase in Rab10 pT73 in VKI (iii, Kruskal–Wallis *p* < 0.0001; Uncorrected Dunn’s Het **p < 0.003, Ho **p < 0.003). **C** Western blot of LRRK2, LRRK2 pS935, GAPDH, Rab10, and Rab10 pT73 in VKI whole brain lysate (i) revealed that MLi2 treatment significantly reduced LRRK2 pS935 in all genotypes (ii, 2-way ANOVA treatment *p* < 0.0001; Uncorrected Fisher’s LSD WT-WTMLi2 ****p < 0.0001; Het-HetMLi2 ***p < 0.0002; Ho-HoMLi2 ****p < 0.0001) and had significant genotype and treatment effects on Rab10 pT73 due to significant reductions in homozygous cells (iii, 2-way ANOVA genotype *p* = 0.04; treatment *p* < 0.009; Uncorrected Fisher’s LSD WT-WTMLi2 *p* = 0.82; Het-HetMLi2 *p* = 0.10; Ho-HoMLi2 **p = 0.007). **D** Western blot of LRRK2, LRRK2 pS935, GAPDH, Rab10, and Rab10 pT73 in whole brain lysate revealed no significant effect of the solubilizing agent Captisol on LRRK2 pS935 (ii, Mann–Whitney *p* = 0.89) or Rab10 pT73 (iii, Mann–Whitney *p* = 0.69). For C.ii–iii, WTCap n = 5, WTMLi2 n = 6, HetCap n = 6, HetMLi2 n = 6, HoCap n = 5, HoMLi2 n = 5

The D620N mutation does not impair VPS35 binding to other retromer complex members (vacuolar protein sorting 26 or vacuolar protein sorting 29; VPS26 & VPS29, respectively) by semi-quantitative co-immunoprecipitation (coIP) in overexpression systems [15, 44–46] or brain lysate from VKI mice [12]. We assayed subunit assembly by coIP in brain lysate from 3-month-old VKI mice and confirmed no genotype effect on the amount of VPS26 pulled by VPS35 (Fig. 1A.i–ii; 1-way ANOVA *p* = 0.44). In cell lines, the D620N mutation impairs VPS35 association with the Wiskott–Aldrich syndrome protein and SCAR homolog (WASH) complex member family with sequence similarity 21 (FAM21) by coIP [44, 45, 47]. We found the level of FAM21 pulled by VPS35 was similarly reduced in coIP of whole-brain lysates from mutant mice (Fig. 1A.i & iii; 1-way ANOVA *p* < 0.003; Uncorrected Fisher’s LSD Het **p < 0.01; Ho **p < 0.01) and we successfully pulled VPS35 by FAM21 in the reverse coIP (Additional file 1: Fig. S1A). In agreement with our previous report and with others [12, 48], we found no genotype effect on the level of VPS35, VPS26, or WASH complex member FAM21 protein relative to β-tubulin in whole brain lysate from 3 month old VKI mice, quantified by WES capillary-based western blot (Additional file 1: Fig. S1B.i–iv).

We performed coIP of striatal lysates pulling for VPS35 and blotting for LRRK2 and for a panel of possible neurotransmitter cargoes relevant to corticostriatal synapses (Additional file 1: Fig. S2A.i). We replicated our previous finding that GluA1 associates with retromer [15] and found two previously unreported putative retromer cargoes; D2-type dopamine receptors (D2R) and GluN1-containing N-methyl-D-aspartate (NMDA)-type glutamate receptors (Additional file 1: Fig. S2A.i). There was no mutation effect on the levels of VPS35, LRRK2, GluN1, D2R, or GluA1 in striatal lysate, nor on their association with VPS35 by coIP (Additional file 1: Fig. S2 A.ii–xi).

Others have reported increased LRRK2 kinase-dependent phosphorylation of Rab10 at threonine 73 (pRab10) in VKI mouse brain in the absence of changes to Rab10 levels [27], a finding we replicated here using fluorescence western blot with knock-out validated phospho-specific antibodies (Fig. 1B.i–iii; Rab10 Kruskal–Wallis p = 0.16; pRab10 Kruskal–Wallis p < 0.0001; Uncorrected Dunn’s Het **p < 0.003, Ho **p < 0.003). We found no difference in LRRK2 protein levels in any genotype (Additional file 1: Fig. S3A.i–iii) and no difference in LRRK2 phosphorylation at the serine 935 residue (pS935, herein pLRRK2) in untreated heterozygous brain. We did find a significant increase of basal pLRRK2 in untreated homozygotes (Additional file 1: Fig. S3A.i–iii), but this was not observed in the larger cohort comparison of vehicle vs. inhibitor treated animals (Fig. 1C). This residue, considered constitutive, is required for LRRK2 kinase activity but is not an autophosphorylation site and its phosphorylation level does not correlate with LRRK2 kinase enzymatic activity levels [49]; however, S935 is dephosphorylated when LRRK2 is inactivated, and it is widely accepted as indicative of effective LRRK2 kinase inhibition [50]. In mice injected with the highly selective LRRK2 kinase inhibitor MLi-2 [27, 41, 49, 50], pLRRK2 was significantly reduced after 2 h (~ 90%) in all genotypes, relative to vehicle injected controls (Fig. 1C.i–ii; 2-way ANOVA treatment *p* < 0.0001; Uncorrected Fisher’s LSD WT-WTMLi2 ****p < 0.0001; Het-HetMLi2 ***p < 0.0002; Ho-HoMLi2 ****p < 0.0001) demonstrating successful target engagement. MLi-2 treatment reversed pRab10 increases in whole brain lysate from VKI mice (Fig. 1C.i & iii; 2-way ANOVA genotype *p* = 0.04; treatment *p* < 0.009; Uncorrected Fisher’s LSD WT-WTMLi2 *p* = 0.82; Het-HetMLi2 *p* = 0.10; Ho-HoMLi2 **p = 0.007) but had little effect on WT brain pRab10 levels, in agreement with Mir et al*.* [27]. This occurred in the absence of any change to levels of LRRK2, GluA1, VPS35, VGluT1, or Rab10 protein (Additional file 1: Fig. S3B.ii–vi). The solubilizing agent sulfobutylether-β-cyclodextrin (Captisol®) had no effect on pLRRK2 or pRab10 compared to saline in western blots of whole-brain lysate from injected mice (Fig. 1D.i–iii; Mann–Whitney *p* = 0.89 & *p* = 0.69, respectively), nor on levels of LRRK2, GluA1, VPS35, VGluT1, or Rab10 (Additional file 1: Fig. S4).

In summary, physiological expression of VPS35 D620N does not alter protein levels of core retromer components, the known interactor FAM21, or LRRK2 and its substrate Rab10, but does result in hyperphosphorylation of Rab10 which is reversed by acute in vivo LRRK2 kinase inhibition. While retromer complex assembly and neuronal cargo binding appear unaltered, we found the association of VPS35 with FAM21 is reduced in VKI brain.

### Increased colocalization and density of endosomal recycling proteins

**Fig. 2:**
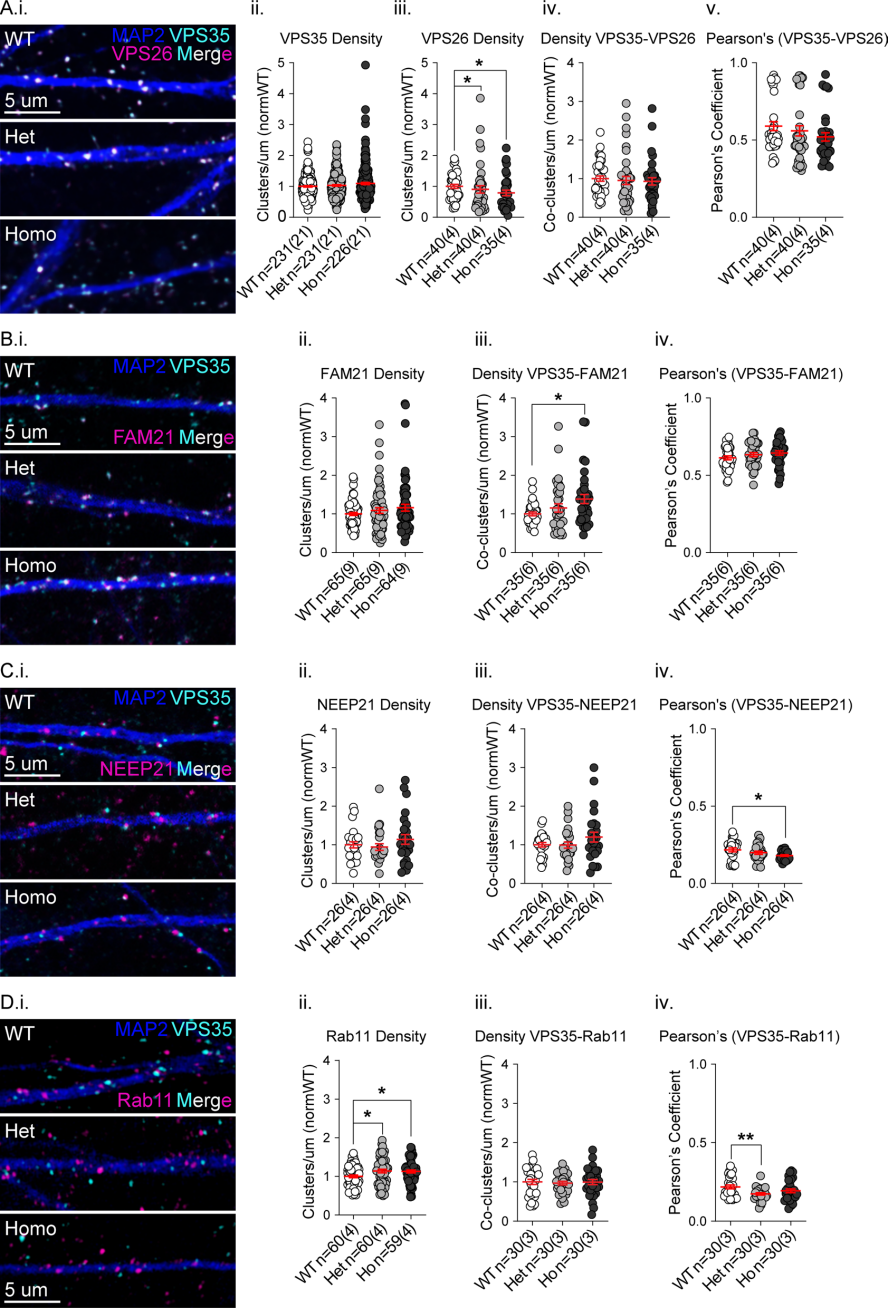
Accumulation of VPS35- FAM21 co-clusters and Rab 11 clusters in VKI dendrites. **A**–**D** Cultured cortical neurons were immunostained for MAP2 (blue), VPS35 (cyan), and VPS26, FAM21, NEEP21, or Rab11 (magenta) **A** VPS35 and VPS26 (i); There was no genotype effect on dendritic cluster density of VPS35 (ii, Kruskal–Wallis *p* = 0.30). VPS26 cluster density was significantly reduced in both mutants (iii, Kruskal–Wallis *p* < 0.04; Uncorrected Dunn’s Het *p < 0.04 & Ho *p < 0.02), yet there was no genotype effect on co-cluster density or Pearson’s coefficient (iv & v, Kruskal–Wallis *p* = 0.45 & 0.10, respectively). **B** VPS35 and FAM21 (i); there was no genotype effect on FAM21 cluster density (ii, Kruskal–Wallis p = 0.24) or Pearson’s coefficients (iv, 1-way ANOVA *p* = 0.24). There was a significant genotype effect on co-cluster density due to increases in homozygous cells (iii, Kruskal–Wallis *p* < 0.03; Uncorrected Dunn’s Ho *p < 0.02). **C** NEEP21 and VPS35 (i); there was no genotype effect on NEEP21 cluster (ii, Kruskal–Wallis *p* = 0.37) or co-cluster density (iii, Kruskal–Wallis *p* = 0.38), but a significant genotype effect on Pearson’s coefficient due to a significant reduction in homozygous VKI dendrites (iv, Welch’s ANOVA *p* < 0.03; Unpaired t with Welch’s correction Ho *p < 0.02). **D** VPS35 and Rab11 (i); Rab11 cluster density was increased in both genotypes (ii, 1-way ANOVA *p* < 0.03; Uncorrected Fisher’s LSD *p < 0.02 & *p < 0.03). There was no genotype effect on co-cluster density (iii, 1-way ANOVA *p* = 0.92), but a significant genotype effect in the Pearson’s coefficient due to reduced correlation in heterozygous dendrites (iv, Welch’s ANOVA *p* < 0.009; Unpaired t with Welch’s correction Het **p < 0.003)

The retromer complex requires the recruitment of the WASH complex to drive the rescue of receptors out of maturing endosomes for recycling to the plasma membrane [51–53]. Since the mutation alters VPS35 interaction with FAM21, we studied the localization of VPS35, its known interactors, and endosomal markers in cortical neuron dendrites by immunostaining at 21 days*-*in-vitro (DIV21) to detect VPS35 colocalization with VPS26 (retromer complex), FAM21 (WASH complex), NEEP21 (early and late endosomes), or Rab11 (recycling endosomes; Fig. 2A–D).

We observed robust colocalization of VPS35 signal with VPS26, a member of the retromer complex core trimer (Fig. 2A.i; Pearson’s ~ 0.6). Dendritic VPS35 cluster density was unaffected by genotype (Fig. 2A.ii; Kruskal–Wallis *p* = 0.30); however, the density of VPS26 clusters was reduced in heterozygous and homozygous neurons (Fig. 2 A.iii; Kruskal–Wallis *p* < 0.04; Uncorrected Dunn’s Het *p < 0.04 & Ho *p < 0.02). There was no genotype effect on colocalized cluster density or Pearson’s correlation coefficient (Fig. 2A.iv–v; Kruskal–Wallis *p* = 0.45 & 0.10, respectively).

There was no genotype effect on the cluster density of the WASH complex member FAM21 (Fig. 2 B.ii; Kruskal–Wallis p = 0.24). VPS35 and FAM21 were robustly colocalized (Fig. 2B.i; Pearson’s ~ 0.6) with no genotype effect on Pearson’s correlation coefficient (Fig. 2B.iv; 1-way ANOVA *p* = 0.24). Contrary to the observed reduction in coIP with FAM21, the density of VPS35-FAM21 co-clusters was increased in mutants in a gene dose-dependent manner, with a significant post hoc increase in homozygous cells (Fig. 2B.iii; Kruskal–Wallis *p* < 0.03; Uncorrected Dunn’s Ho *p < 0.02).

Neuronal endosomal enriched protein 21 (NEEP21 or NSG1) is itinerant in dendritic endosomes and rapidly degraded with little to no recycling [54]. We therefore used NEEP21 as a marker of the non-recycling endolysosomal pathway (Fig. 2C.i). We observed no genotype effect on the density of NEEP21 clusters (Fig. 2C.ii; Kruskal–Wallis *p* = 0.37), nor VPS35-NEEP21 co-cluster densities (Fig. 2C.iii; Kruskal–Wallis *p* = 0.38), suggesting no change in VPS35 localization at early or late endosomes. VPS35 and NEEP21 were tightly apposed with little overlap (Fig. 2C.i; Pearson’s coefficient ~ 0.2), in accordance with retromer-coated tubules extending from endosomes to drive recycling, not lysosomal degradation. Consistent with this, there was a decrease in the Pearson’s coefficient in VKI dendrites (Fig. 2C.iv; Welch’s ANOVA *p* < 0.03; Unpaired t with Welch’s correction Ho *p < 0.02).

Rab11 decorates recycling endosomes (REs) and participates in AMPAR surface trafficking [55–60], thus we used Rab11 as a marker of REs (Fig. 2D.i). Rab11 cluster density was increased in both VKI mutants (Fig. 2D.ii; 1-way ANOVA *p* < 0.03; Uncorrected Fisher’s LSD *p < 0.02 & *p < 0.03). Despite increased Rab11 cluster abundance, there was no genotype effect on the colocalization density of VPS35 and Rab11 (Fig. 2D.iii; 1-way ANOVA *p* = 0.92) and a decrease in Pearson’s coefficient in heterozygous cells (Fig. 2D.iv; Welch’s ANOVA *p* < 0.009; Unpaired t with Welch’s correction Het **p < 0.003). A reduction in Pearson’s coefficients in this instance may be reflective of an accumulation of recycling endosomes downstream of retromer tubulation.

In summary, the D620N mutation increased clustering of proteins found on structures involved in surface delivery (Rab11) and VPS35 co-clustered with complexes that drive surface recycling (FAM21). Conversely, overlap of VPS35 signal with a marker of the degradative pathway (NEEP21) was reduced. Together the observations suggest the mutation increases capacity for surface recycling; alternatively it may halt recycling traffic, resulting in the accumulation of surface bound carriers (VPS35-FAM21 & Rab11).

### Glutamate transmission is increased in VKI and not reversed by LRRK2 kinase inhibition

To help determine if mutation effects reported in Fig. 2 reflect an increase in, or backlog of, dendritic surface traffic, we examined glutamatergic synapse function in cortical cultures from VKI mice.

**Fig. 3:**
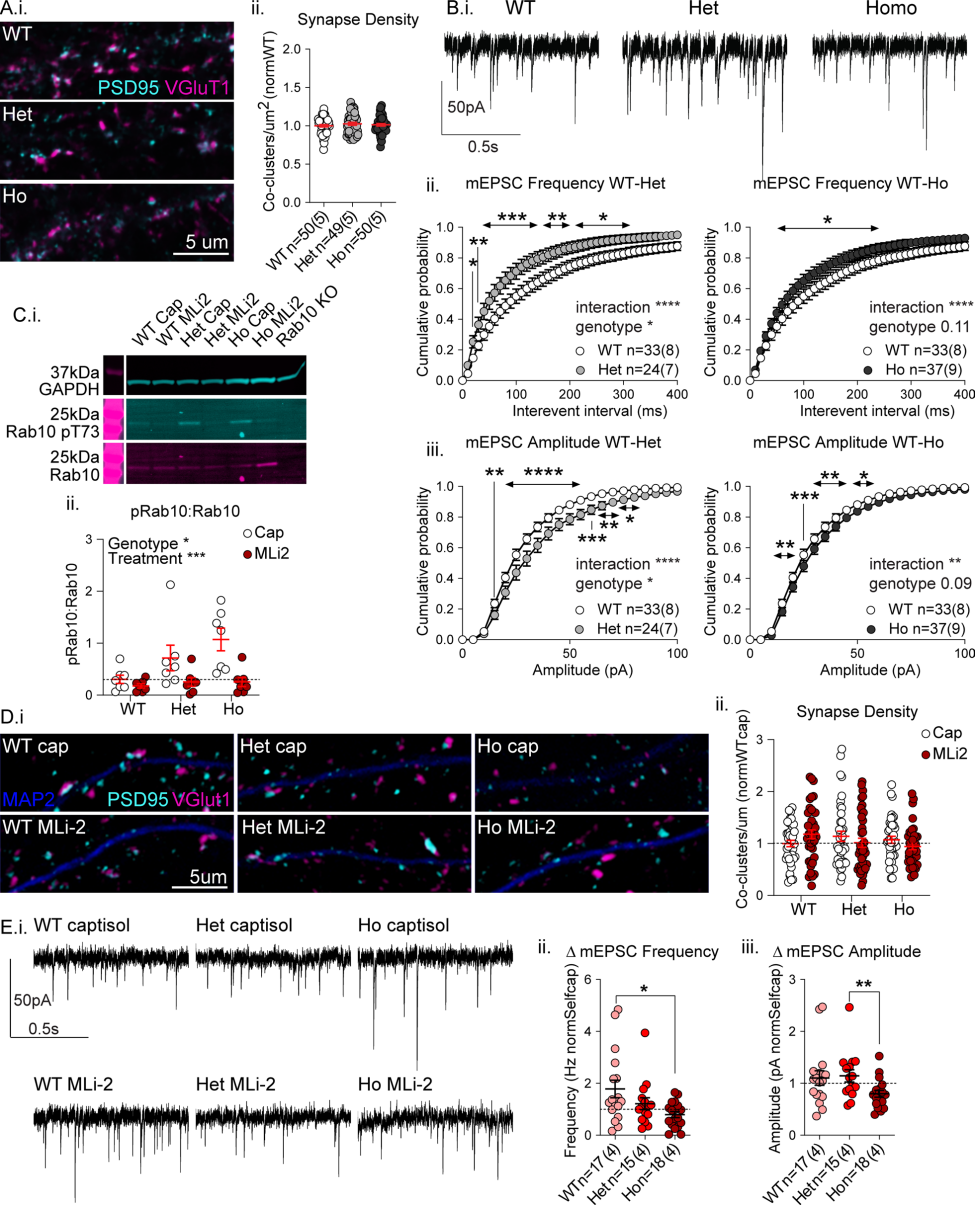
Synaptic transmission is increased in VKI and altered by acute LRRK2 kinase inhibition. **A** Cortical neurons immunostained for PSD95 (cyan) and VGluT1 (magenta) (i). There was no genotype effect on VGluT1-PSD95 co-clusters (ii, 1-way ANOVA *p* = 0.57). **B** Whole-cell patch voltage clamp recording of mEPSCs in cortical neurons (i). There were significant shifts in frequency cumulative probabilities due to significantly smaller inter-event intervals in cells from both genotypes (ii, 2-way RM ANOVA WT-Het interaction *p* < 0.0001; genotype *p* < 0.03; 2-way RM ANOVA WT-Ho interaction *p* < 0.0001; genotype *p* < 0.11; Uncorrected Fisher’s LSD ***p < 0.001; **p < 0.007; *p < 0.05). Significant shifts were seen in amplitude cumulative probabilities due to increases in heterozygous and homozygous cells (iii, 2-way RM ANOVA WT-Het interaction *p* < 0.0001; genotype *p* < 0.03; 2-way RM ANOVA WT-Ho interaction *p* < 0.002; genotype *p* < 0.09; Uncorrected Fisher’s LSD ****p < 0.0001; ***p < 0.004; **p < 0.01; *p < 0.05). **C** Western blot of MLi-2 or vehicle treated cortical culture lysates probed for Rab10, Rab10 pT73, and GAPDH (i). There were significant genotype and treatment effects on Rab10 pT73 due to significant increases in vehicle-treated homozygous neurons over WT, which are significantly reduced by treatment (ii, 2-way ANOVA genotype x treatment *p* < 0.08; genotype *p* < 0.03; treatment *p* < 0.0004; Uncorrected Fisher’s LSD WT-Ho *p < 0.01; WT-WTMLi2 *p* = 0.99; Het-HetMLi2 *p* = 0.26; Ho-HoMLi2 **p < 0.005). **D** Cortical neurons immunostained as in A) plus MAP2 (blue) following MLi-2 or vehicle treatment (i). There were no significant effects on PSD95-VGluT1 co-cluster density (ii, 2-way ANOVA genotype x treatment *p* = 0.08; genotype *p* = 0.52; treatment *p* = 0.68; All groups n = 40[4]). **E** Whole-cell patch voltage clamp recording of mEPSCs in cortical neurons following MLi-2 treatment (i). Change in mEPSC frequency following treatment revealed a significant genotype effect due to a ~ two-fold increase in mEPSC frequency in only WT cells following treatment (ii, Kruskal–Wallis *p* < 0.04; Uncorrected Dunn’s WT-Ho **p < 0.01). Change in mEPSC amplitude following treatment revealed a significant genotype effect due to mildly opposing effects in heterozygous and homozygous cultures (iii, Kruskal–Wallis *p* < 0.03; Uncorrected Dunn’s Het-Ho **p < 0.01)

In light of reports that VPS35 knock-down and overexpression both result in impaired neurite outgrowth [17, 19], we performed Sholl analysis of cultured cortical neurons from VKI expressing green fluorescent protein (pAAV-CAG-GFP) at DIV21 and found no mutant effects on cell density or neurite morphology (Additional file 1: Fig. S5). We previously reported that VPS35 wild-type and mutant overexpression reduced synapse number and miniature excitatory post-synaptic current (mEPSC) frequency in cultured cortical neurons [15]. Here excitatory synapses were quantified by immunostaining for presynaptic and postsynaptic markers, vesicular glutamate transporter 1 (VGluT1) and postsynaptic density protein 95 (PSD95; Fig. 3A.i), respectively. There was no genotype effect on density of synapses, as evidenced by equal numbers of VGluT1-PSD95 co-clusters (Fig. 3A.ii; 1-way ANOVA *p* = 0.57) and individual cluster densities of PSD95 and VGluT1 (Additional file 1: Fig. S6A.i–ii). In concert with equivalent neurite morphology (Additional file 1: Fig. S5), the data suggest no difference in the density of glutamatergic synapses in VKI cultures. That said, in homozygous neurons, VGluT1 cluster intensity was decreased (Additional file 1: Fig. S6 A.iii), possibly reflecting a presynaptic effect in the homozygous mutant.

To assess glutamate synapse function, we performed whole-cell patch clamp recording of mEPSCs in VKI cultures (Fig. 3B.i). Inter-event interval cumulative probabilities showed that event frequency is higher in mutant neurons (Fig. 3B.ii; 2-way RM ANOVA WT-Het interaction *p* < 0.0001; genotype *p* < 0.03; 2-way RM ANOVA WT-Ho interaction *p* < 0.0001; genotype *p* < 0.11). Increased mEPSC frequencies on a background of equivalent synapse density is usually interpreted as increased probability of vesicular release (Pr), or fewer silent (AMPAR-lacking) synapses.

In our previous report, we found mutant overexpression resulted in larger mEPSC amplitudes than WT; however, neither were significantly different from control making it difficult to determine whether this represented a gain- or loss-of-function upon surface AMPAR trafficking [15]. Here we found the amplitude of mEPSCs was increased in mutant neurons (Fig. 3B.iii; 2-way RM ANOVA WT-Het interaction *p* < 0.0001; genotype *p* < 0.03; 2-way RM ANOVA WT-Ho interaction *p* < 0.002; genotype *p* < 0.09). Changes in amplitude are usually taken to reflect increased surface expression of AMPARs, or altered receptor subtype expression i.e., a higher percentage of GluA2-lacking AMPARs with a higher channel conductance and faster decay kinetics (reviewed in: 61, 62). Thus, we examined mEPSC decay tau and single-channel conductance by peak-scaled non-stationary fluctuation analysis (NSFA) but found no significant genotype effect on either, suggesting a similar AMPAR subunit composition (Additional file 1: Fig. S6B.i–iii). Thus, we conclude increased mEPSC amplitude is due to increased AMPAR surface levels.

Glutamate synaptic phenotypes in LRRK2 G2019S knock-in mice are reversed by acute LRRK2 kinase inhibition [35]. Thus, we hypothesized that in light of increased LRRK2 kinase activity in VKI brain (Fig. 1), LRRK2 inhibition by MLi-2 would reverse glutamatergic phenotypes in VKI primary cortical cultures. We treated DIV21 primary cortical cultures for 2 h with 500 nM MLi-2. LRRK2 and pLRRK2 were detected in culture lysates by fluorescent western blot, and pLRRK2 appeared reduced after MLi2 in all genotypes, although we deemed bands were too close to background to quantify reliably (Additional file 1: Fig. S7). Due to its large size (286 kDa), LRRK2 is difficult to assay by western blot, usually requiring a high concentration of total protein for detection with currently available antibodies. As a proxy for LRRK2 activity in vitro, we assayed pRab10 and found a mutation dose-dependent increase in pRab10 (as seen in brain in Fig. 1). MLi-2 treatment reduced pRab10 in all genotypes and significantly reduced VKI pRab10 hyperphosphorylation levels to that of WT (Fig. 3 C.i–ii; 2-way ANOVA genotype x treatment *p* < 0.08; genotype *p* < 0.03; treatment *p* < 0.0004; Uncorrected Fisher’s LSD WT-WTMLi2 *p* = 0.99; Het-HetMLi2 *p* = 0.26; Ho-HoMLi2 **p < 0.005). Thus, D620N results in increased pRab10 in cultured cortical neurons at DIV21 (as in brain Fig. 1), and is reversed by LRRK2 inhibition with MLi-2.

The effect of LRRK2 kinase inhibition on excitatory synapses was first examined by immunostaining for PSD95 and VGluT1 to determine synapse density (Fig. 3D.i). There were no significant effects of genotype or treatment on excitatory synapse density (Fig. 3D.ii; 2-way ANOVA genotype × treatment *p* = 0.08; genotype *p* = 0.52; treatment *p* = 0.68), despite a trend to increases in MLi-2 treated WT neurons relative to vehicle-treated control.

To assay the effects of MLi-2 treatment on synapse function, we conducted whole-cell patch clamp recording of mEPSCs (Fig. 3E.i). We observed an unexpected near twofold increase in mEPSC frequency in treated WT cells and little effect in heterozygous or homozygous neurons (Fig. 3E.ii; Kruskal–Wallis *p* < 0.04; Uncorrected Dunn’s WT-Ho **p < 0.01). MLi-2 increased mEPSC amplitude slightly in WT and heterozygous cells and produced a significantly different effect (a reduction) in homozygous cells relative to heterozygous neurons (Fig. 3E.iii; Kruskal–Wallis *p* < 0.03; Uncorrected Dunn’s Het-Ho **p < 0.01).

Together the data demonstrate the D620N mutation results in increased mEPSC frequency and amplitude, in the absence of changes to synapse density or neurite complexity. This suggests an increase in surface expression of synaptic AMPARs (mEPSC amplitude) and either an increase in the probability of quantal glutamate release, or an increase the number of excitatory synapses with functional surface AMPARs (mEPSC frequency). Acute treatment with MLi-2 reduced LRRK2 substrate phosphorylation with little or no effect on synapse density and had minor effects on mEPSC amplitude. MLi-2 did not rescue elevated frequency in mutants, but resulted in a near two-fold increase in mEPSC frequency in wild-type cells that could reflect either an increase in glutamate release, or increased delivery of AMPARs to AMPAR-lacking (silent) synapses.

### Surface GluA1 is increased basally in VKI and increased in WT by LRRK2 kinase inhibition

**Fig. 4:**
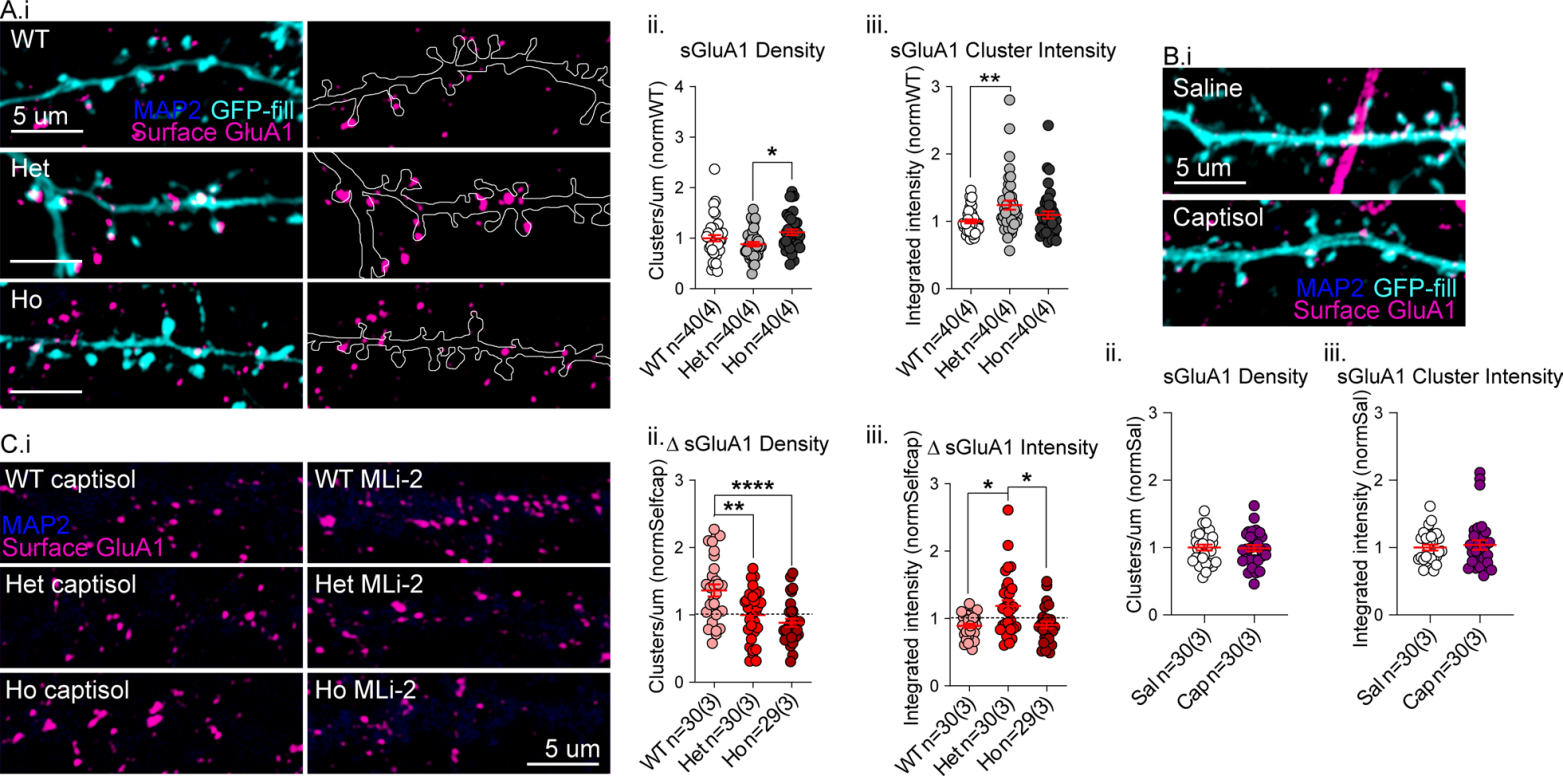
Surface GluA1 is increased in VKI and altered by acute LRRK2 kinase inhibition. **A** GFP-filled (cyan) cultured cortical cells immunostained for MAP2 (blue; to ensure no permeabilization) and surface GluA1 (magenta) (i, left panel); in silico neurite outlines with only GluA1 staining displayed (i, right panel). There was a significant genotype effect on GluA1 cluster density, due to opposing effects on heterozygous and homozygous cells (ii, Kruskal–Wallis *p* < 0.009; Uncorrected Dunn’s Het-Ho *p < 0.05). There was a significant genotype effect on surface GluA1 cluster intensity (synaptic GluA1) due to significant increases in heterozygous cells (iii, Kruskal–Wallis *p* < 0.007; Uncorrected Dunn’s Het **p < 0.02). **B** GFP-filled cultured cortical neurons from WT mice treated with saline or Captisol and immunostained for MAP2 (blue), GFP (cyan), and extracellular GluA1 (magenta) without permeabilization. There were no effects of Captisol treatment on surface GluA1 cluster density (ii, Unpaired t-test *p* = 0.87) or intensity (iii, Mann–Whitney *p* = 0.73). **C** Non-permeabilized cultured cortical cells immunostained for MAP2 (blue), and surface GluA1 (magenta) following acute MLi-2 or vehicle treatment (i). Changes to GluA1 surface density after treatment showed a significant genotype effect due to increases in WT cells not observed in heterozygous and homozygous mutant cells (ii, Welch’s ANOVA *p* < 0.0002; Unpaired t with Welch’s correction WT-Het **p < 0.003; WT-Ho ****p < 0.0001). There was a significant genotype effect on change in surface GluA1 intensity following treatment, due to opposing increases in heterozygous cells and decreases in WT and homozygous cells (iii, Kruskal–Wallis *p* < 0.02; WT-Het *p < 0.02; Het-Ho *p < 0.02)

In the face of similar synapse numbers, increased mEPSC frequency can result from increased quantal glutamate release, or unsilencing of AMPA-silent synapses through surface delivery of AMPARs. To disambiguate the source of the observed mEPSC frequency changes, and to confirm that changes to mEPSC amplitude are the result of changes to synaptic AMPAR expression, we performed immunostaining against an extracellular epitope of the AMPAR subunit GluA1 in non-permeabilized neurons (Fig. 4A.i). We targeted GluA1 as we previously demonstrated that VPS35 preferentially associates with this AMPAR subunit by coIP in mouse brain lysate [15]. Neither VKI culture had surface GluA1 cluster densities significantly different from WT; however, heterozygous neurons had significantly fewer clusters than homozygous (Fig. 4A.ii; Kruskal–Wallis *p* < 0.009). Thus mEPSC frequency changes, at least in heterozygous neurons, likely reflect an increase in Pr of glutamate at excitatory synapses. Surface GluA1 cluster intensity was higher in both mutants, and significantly so for heterozygous neurons in multiple comparisons, mirroring the increases in mEPSC amplitude (Fig. 4A.iii; Kruskal–Wallis *p* < 0.007; Uncorrected Dunn’s Het **p < 0.02). The mutation does not affect GluA1 protein expression or association with VPS35 by coIP in cortical lysate (Additional file 1: Fig. S8A–B) or striatal lysate (Additional file 1: Fig. S2), nor does it affect colocalization density of VPS35 with GluA1 in cultured cortical neuron dendrites by immunocytochemistry (Additional file 1: Fig. S8 C.i,iii,iv). We did find a significant reduction in total dendritic GluA1 clusters that were not colocalized with VPS35 in homozygous neurons, possibly revealing an additional postsynaptic phenotype similar to the presynaptic reduced VGluT1 intensity in the homozygous mutant (Additional file 1: Fig. S8C.ii).

We also performed surface GluA1 staining on non-permeabilized cultured cortical neurons from WT mice at DIV21 following 2 h of treatment with saline or Captisol® (Fig. 4B.i) to ensure no vehicle effects on AMPAR expression. We found no effect of Captisol® on either the density (Fig. 4B.ii; Unpaired t-test *p* = 0.87) or intensity (Fig. 4B.iii; Mann–Whitney *p* = 0.73) of clusters, suggesting that the vehicle does not affect GluA1 surface expression.

Surface GluA1 staining was then performed on cultured VKI cortical neurons following acute treatment with MLi-2 or vehicle (Fig. 4C.i). Surface GluA1 cluster density revealed MLi-2 produced a ~ 50% increase in WT cluster density, and little effect in mutants (Fig. 4C.i&ii; Welch’s ANOVA *p* < 0.0002; Unpaired t with Welch’s correction WT-Het **p < 0.003; WT-Ho ****p < 0.0001). The effect of MLi-2 on surface GluA1 cluster density and mEPSC frequency in WT cells (with a slight increase in excitatory synapse density) suggests LRRK2 kinase inhibition drives delivery of AMPARs to a small number of new synapses and more so to existing AMPAR-lacking synapses. MLi-2 treatment resulted in different effects on surface GluA1 cluster intensity in heterozygous neurons (increase) relative to a small decrease observed in WT and homozygous cells (Fig. 4C.iii; Kruskal–Wallis *p* < 0.02; WT-Het *p < 0.02; Het-Ho *p < 0.02). The data suggest a genotype-dependent effect of LRRK2 kinase inhibition on AMPAR surface expression, which is similar to MLi-2 effects on mEPSC amplitude.

The LRRK2 kinase substrate Rab10 is involved in actin dynamics at recycling endosomes [63], transport of retromer cargoes including GLR-1 [64–68], and can be co-purified with synaptic vesicles [69]. Given that pRab10 was increased in mutant cultures (Fig. 3) and that MLi-2 affected surface AMPARs (Fig. 4), we analyzed dendritic Rab10 signals and colocalization with GluA1 in cultured cortical neurons at DIV21 by immunocytochemistry (Additional file 1: Fig. S9A.i). We found Rab10 clusters were present along dendrites, but sparse compared to other dendritic endosome markers. We saw increased Rab10 cluster density in both mutants that strongly trended to significance (Additional file 1: Fig. S9A.ii); however, GluA1-Rab10 were poorly colocalized (Additional file 1: Fig. S9A.i; Pearson’s 0.17) and there were no significant genotype effects on co-cluster density or Pearson’s coefficients (Additional file 1: Fig. S9A.ii–iii). The data demonstrate that Rab10 clusters may be increased along VKI dendrites, but do not provide any evidence that Rab10 is involved in local surface delivery or recycling of AMPARs within dendrites.

Altogether the data demonstrate an endogenous VPS35 D620N mutation produces a gain-of-function in pre- and post-synaptic glutamate transmission. Synaptic surface AMPAR expression (increased surface GluA1 cluster intensity and mEPSC amplitude) and the Pr of glutamate at excitatory synapses (increased mEPSC frequency with no change in synapse or surface GluA1 cluster density) are increased. Homozygous neurons had an additional phenotype of reduced VGluT1 intensity and dendritic GluA1 cluster density that may account for the less-pronounced increase to synaptic transmission we observed. MLi-2 treatment had the greatest effect on post-synaptic AMPAR expression in WT cells, through elevated delivery of GluA1-containing AMPARs to AMPAR-lacking silent synapses (increased mEPSC frequency and surface GluA1 cluster density with minimal changes to synapse density). This effect was absent in mutant cells. The abundance of GluA1 in existing AMPAR-containing synapses, as measured by surface GluA1 cluster intensity, was altered by MLi-2 treatment in a genotype-dependent manner. The clearest change was an increase in synaptic GluA1 in heterozygous neurons, which are genetically the most relevant to human VPS35 mutation carriers.

## Discussion

Previous reports of VPS35 neurobiology at glutamatergic synapses, and the effects of the D620N mutant, have relied on exogenous protein expression or knock-out, and produced somewhat conflicting results. Studies on retrograde trafficking of the canonical retromer cargo cation-independent mannose-6-phosphate receptor (CI-MPR), for example, concluded that the mutation results in a loss-of-function [15, 44, 46, 47] or has no effect [45, 70]. This discordance may result from the variety of cell types and modes of expression used to glean insights. We previously reported that exogenous WT VPS35 expression in mouse cortical neurons resulted in a slight decrease in mEPSC amplitudes compared to control, whereas mutant expression resulted in a slight increase. The result was a significant increase in mEPSC amplitudes in neurons expressing mutant D620N protein [15]. The mutation also reduced motility of exogenous GFP-tagged VPS35 in dendrites and its localization to spines—results we interpreted at the time as potentially indicative of a loss-of-function [15]. The only other study to date on the effect of D620N on AMPAR trafficking found exogenous D620N expression in hippocampal neurons from haploinsufficient mice was unable to rescue impairments in LTP [21].

Having found endogenous expression of VPS35 D620N increases surface GluA1 expression and mEPSC amplitude in cortical neurons from VKI, we conclude this may be a gain-of-function effect which is only evident with endogenous total levels of VPS35. Overexpression of VPS35 has similar effects as haploinsufficiency or knock-down on neurite outgrowth and synapse density [15–17, 19, 20, 24, 71]. This implies that overexpressing VPS35 may have a dominant-negative effect on some of its functions. An emerging theme in the literature is that changing ratios of VPS35 to its multiple binding partners can shift its function, thus it follows that its function is sensitive to both under and overexpression. Our work here underscores the importance of endogenous protein expression levels when assaying the physiological function of VPS35 and any mutant effects.

**Fig. 5:**
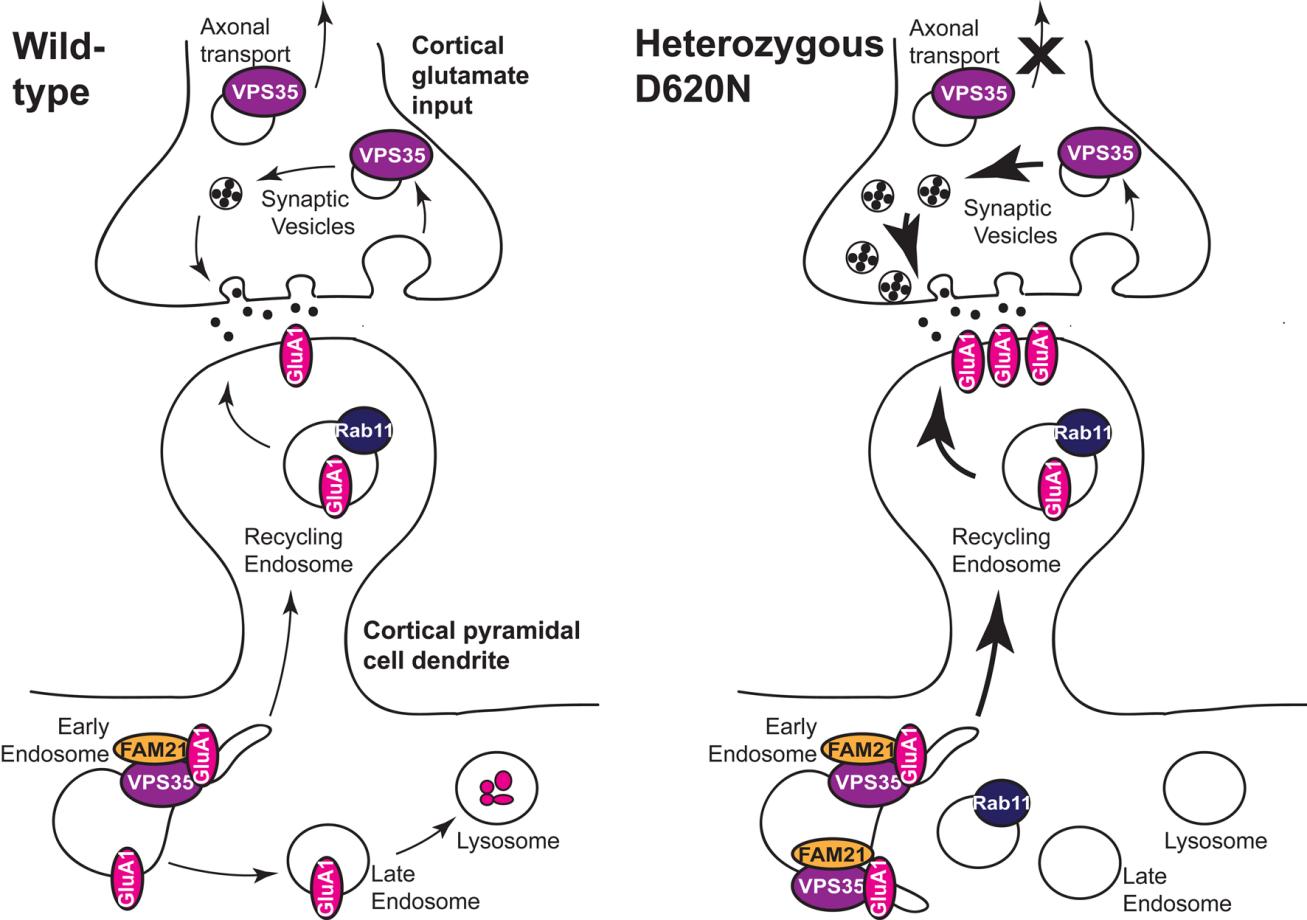
Working model of increased surface AMPAR expression in D620N heterozygous mutants. In wild-type cells, postsynaptic VPS35 traffics GluA1-containing AMPARs. At the drosophila neuromuscular junction, VPS35 localizes to large endocytic structures resembling bulk-endocytosed membrane, raising the possibility that it participates in SV regeneration [25]; however, in mammalian neurons it localizes to only a subset of terminals [22] and retromer deficiency has no effect on SV endo- or exocytosis [22] or neurotransmitter release [16, 21]. Thus, we propose that presynaptic VPS35 may participate in recycling or retrograde transport of presynaptic receptors, channels, and/or SV proteins. In heterozygous neurons, there is increased abundance of endosomal structures positive for VPS35 and FAM21, accumulation of Rab11 + ve recycling endosomes, and increased surface expression of GluA1, thus we propose that the D620N mutation causes increased surface recycling of GluA1. Given the proposed presynaptic functions of retromer, we hypothesize that the observed increase in the probability of glutamate release is the result of either increased SV regeneration by retromer, or complex alterations to the recycling and axonal trafficking of presynaptic proteins by retromer

A reduction in the association of FAM21 with VPS35 D620N has been proposed elsewhere as a mechanism for mutation effects on retrograde trafficking of CI-MPR [44, 47] and autophagy [45]. While we show here (for the first time) that this reduced association also occurs in whole brain lysates of endogenous knock-in animals, this impairment phenotype is not obviously related to GluA1 trafficking. A recent study showed that reduced association of VPS35 D620N with FAM21 can be rescued by TBC1D5 knock-down [72], thus the loss of association is likely not due to an inherent loss of VPS35’s ability to bind FAM21 in the presence of the mutation. Furthermore, loss of FAM21 by knock-down has been shown to cause misdirection of surface-bound retromer cargoes to the TGN, reducing their surface expression [53]. Our results are suggestive of increased retromer association with FAM21 in dendrites, and increased surface trafficking of GluA1 in VKI pyramidal cortical neuron dendrites (Fig. 5). The reduced association of VPS35 with FAM21 observed in brain tissue may be relevant for other retromer cargoes/trafficking pathways in neuronal soma, or in non-neuronal cell types.

To date, little is known about retromer in presynaptic physiology. Retromer is highly motile in axons, and present in glutamatergic synaptic boutons in cultured murine neurons [15, 22], but retromer deficiency or acute knock-down in murine hippocampal slices has little or no effect on synaptic glutamate release [16, 21], or SV endo- or exocytosis [22]. We previously found expression of both WT and mutant VPS35 in cortical neurons resulted in fewer excitatory synapses and reduced frequency of mEPSCs, but there were no clear mutant-specific effects on frequency, and only a trend toward synapse density differences in mutant-expressing cells [15]. It is important to note this was a fairly low expression level, on the background of WT protein. Therefore, asides our observations of increased mEPSC frequency in VPS35 mutants here, there is little other evidence to date for retromer having a critical role in glutamate release per se. VPS35 associates with presynaptically expressed transmembrane proteins such as D2R (present study) and dopamine transporter (DAT; 73), and dopamine release is increased in ex vivo slices from 3-month-old VKI mice alongside changes in expression of DAT and vesicular monoamine transporter 2 (VMAT2; 48); thus it may impact upon SV release indirectly by participating in the sorting and recycling or retrograde transport of old synaptic vesicle proteins, presynaptic channels, and/or receptors (Fig. 5). Whether retromer is involved in transmitter release directly, or indirectly by regulating axonal transport and/or recycling of synaptic proteins that modulate release, requires further study.

While much remains to be discovered about a presynaptic role for VPS35, it is noteworthy that PD-associated mutations in LRRK2 cause similar increases in glutamate and dopamine release [30, 35, 74, 75] in addition to impinging upon several post-synaptic processes [36–38]. Pathogenic mutations in LRRK2 clearly increase its kinase function [39, 41, 76], resulting in increased phosphorylation of several LRRK2 substrates including a large subset of Rab-GTPases that have roles in the SV cycle and postsynaptic AMPAR traffic [41, 42, 77]. Previous studies have shown that acute (30 min) treatment with LRRK2 kinase inhibitors is sufficient to reverse transmitter release and post-synaptic phenotypes in LRRK2 G2019S mice [35, 37].

Our replication of the previous report of D620N mutation resulting in LRRK2 kinase-dependent hyperphosphorylation of Rab10 [27] led us to hypothesize that glutamate transmission phenotypes in VKI would be reversed by acute LRRK2 kinase inhibition. The results suggest the mechanism of glutamate dysregulation in VKI cultures is distinct from that in LRRK2 G2019S mutant mice (in which glutamate release phenotypes are reversed within 30 min of LRRK2 kinase inhibition; 35,37). It is possible VKI phenotypes are unrelated to increased LRRK2 kinase activity or Rab10 phosphorylation state; however, it remains possible that longer-term inhibition will render different results. LRRK2 localizes to endosomes [78], co-immunoprecipitates with VPS35 in brain lysates (in the present study and elsewhere [24, 28]), and has been shown to interact functionally with VPS35 in neurons [24, 25]. Thus, it remains a possibility that LRRK2 is involved in the dysregulation of glutamate synapses in VKI through one of its other functional domains (e.g., structural scaffolding or GTPase activity). Future investigations of the contributions of the other functional domains of LRRK2 in VPS35 D620N synaptic phenotypes may prove illuminating.

Previous work in LRRK2 knock-out mice has shown that LRRK2 negatively regulates protein kinase A (PKA)-mediated AMPAR insertion, can alter PKA trafficking in and out of spines, and that pathogenic mutations in LRRK2 increase PKA-mediated phosphorylation of GluA1 [36]. While Parisiadou and colleagues [36] found that forskolin-induced phosphorylation of GluA1 by PKA was resistant to LRRK2 kinase inhibition, they did not assay the effect of LRRK2 inhibition on endogenous GluA1 phosphorylation nor surface expression. Here we show that LRRK2 kinase activity negatively regulates trafficking of GluA1-containing AMPARs to silent synapses in developing neurons, yet has no effect on GluA1 surface expression in mutant neurons. This could be indicative of accelerated synapse maturation in mutant cells beyond a LRRK2-sensitive time-point. While outside the scope of the current study, further inquiry into the time course of synapse formation and maturation in VKI neurons, and the role of LRRK2 and its kinase activity in PKA-dependent and -independent AMPAR trafficking, is warranted.

To our knowledge this is the first study of glutamatergic neuron biology in a knock-in model of VPS35 D620N parkinsonism. We found that the D620N mutation results in increases to glutamate release similar to LRRK2 G2019S, with an additional post-synaptic phenotype of increased GluA1 surface expression (Fig. 5). LRRK2 kinase inhibition reversed pRab10 increases in mutant brain and cultures, but did not reduce postsynaptic AMPAR expression in heterozygous cells (that are most relevant to human PD), or mutant Pr; however, LRRK2 inhibition increased forward traffic of GluA1 to silent synapses in WT. This suggests caution is necessary in the wider application of LRRK2 kinase inhibitor treatment for PD e.g., non-LRRK2 PD. We add support to the hypothesis that synaptic transmission is augmented at early time points in PD, which potentially represents early pathophysiological processes that can be targeted to prevent transition to later pathological damage (reviewed in: 40,79,80).

## Methods

### VPS35 D620N knock-in mice and genotyping

Constitutive VPS35 D620N knock-in mice (VKI) were generated by Ozgene (Australia) under guidance of Dr. Matthew Farrer using gene targeting in C57Bl/6 embryo stem cells (Bruce4) as previously described [48]. The VKI strain has been deposited in Jackson Labs with open distribution supported by the Michael J Fox Foundation (*VPS35* knock-in: B6(Cg)-Vps35tm1.1Mjff/J). All mice were bred, housed, and handled according to Canadian Council on Animal Care regulations. All procedures were conducted in accordance with ethical approval certificates from the UBC ACC (A16-0088; A15-0105) and the Neuro CNDM (2017-7888B). Animals were group-housed in single-sex cages with littermates after weaning.

The procedure for creating the VKI resulted in 51 base-pair insertion in the non-coding regions of *VPS35*, such that PCR amplification of the WT gene using appropriate primers creates a product of 303 base pairs and the knock-in gene a product of 354 base pairs. The animals were genotyped by PCR amplification of *VPS35*, followed by confirmation of the presence of a 303 bp product (WT), a 354 bp product (Ho) or both (Het). Small tissue samples were digested in 100 uL 10% Chelex (Bio-Rad 142–1253) at 95 ℃ for 20 min and spun down to result in DNA-containing supernatant. 2uL DNA was mixed with 18uL of master mix containing taq polymerase, buffer (DNAse- and RNAse-free water, 10 × buffer, MgCl 25 mM, dNTPs 10 mM), and primers (ThermoFisher Custom DNA oligos: forward-TGGTAGTCACATTGCCTCTG; reverse-ATGAACCAACCATCAATAGGAACAC) according to the instructions for the taq polymerase kit (Qiagen 201203), and the PCR was performed in a programmable machine (program available upon request). Agarose gel electrophoresis was used to separate the products on a 3–4% gel with fluorescent DNA dye (ZmTech LB-001G) and visualized on a Bio-Rad ultraviolet gel imager.

### Western blots and co-immunoprecipitations

Three-month-old male mice were decapitated, and brains removed and chilled for 1 min in ice-cold carbogen-bubbled artificial cerebrospinal fluid (ACSF; 125 mM NaCl, 2.5 mM KCl, 25 mM NaHCO3, 1.25 mM NaH2PO4, 2 mM MgCl2, 2 mM CaCl2, 10 mM glucose, pH 7.3–7.4, 300–310 mOsm). For region-specific analysis, this was followed by rapid (< 6 min) microdissection of cortex, striatum, hippocampus, dorsal midbrain, olfactory bulbs, and cerebellum, with all remaining tissue pooled as ‘rest’. Tissues were flash frozen in liquid nitrogen and either lysed for immediate use or stored at −80 ℃. For *WES* and chemiluminescent western blots, tissues were mechanically homogenized in HEPES buffer (20 mM HEPES, 50 mM KAc, 200 mM Sorbitol, 2 mM EDTA, 0.1% Triton X-100, pH 7.2; Sigma Aldrich) containing protease inhibitor cocktail (Roche 11697498001), then incubated on ice for 45 min with occasional gentle agitation. For fluorescent western blots, tissue was homogenized by probe sonication at 20 kHz for 10 s in ice-cold TBS buffer (tris-buffered saline, 1% Triton X-100; pH 7.4) containing protease inhibitor (Roche 11697498001) and phosphatase inhibitor (Sigma 4906845001) cocktails. Protein was quantified by Pierce BCA assay (ThermoFisher 23255) and samples were adjusted to equal concentrations in lysis buffer prior to denaturing.

For traditional chemiluminescence detection, 10–15 µg protein was prepared in 4× NuPage LDS sample buffer (Invitrogen NP0008) with 2.5% β-mercaptoethanol or 500 mM DTT to a total volume of 10–24 uL, and denatured at 70 °C for 10 min. Samples were loaded into a NuPAge 4–12% Bis–Tris gel (Invitrogen NP0322BOX) in an XCell II Blot module (Invitrogen) and run at 70 V for 30 min, followed by 110 V for 1 h. Separated proteins were transferred to methanol-activated Immobilon-P PVDF membrane (Millipore IPVH00010) for 90 min at 25 V at room temperature, then blocked with 5% milk in PBS for 1 h at room temperature. Membranes were probed by shaking with primary antibodies in primary antibody solution (PBS, 2% BSA, 0.05% Tween-20) for either 1 h at room temperature or overnight at 4 °C, washed 4 × in PBST (PBS, 0.05% Tween-20), and detected by HRP-conjugated secondary antibodies (Invitrogen; shaken in PBS, 5% milk, 0.05% Tween-20 for 30 min at room temperature). Chemi-luminescence was detected with Pierce ECL (ThermoFisher 32209) imaged on a Chemi-Doc imaging system (Cell-Bio).

Fluorescence western blots were performed with the following modifications to the above protocol: (1) 40 ug protein samples; (2) Bolt 4–12% Bis–Tris Plus Gels (Invitrogen NW04120BOX); (3) Fluorescence detection optimized membrane, Immobilon-FL PVDF (Millipore IPFL00010); (4) TBS was substituted for PBS in all buffers to prevent possible cross-reaction of phospho-specific antibodies; (5) transfer increased to 2.5 h for larger proteins (LRRK2), and; (6) LiCor fluorescent secondary antibodies were used (LI-COR) and imaged on a LI-COR Odyssey Infrared imaging system (LI-COR).

When necessary, phosphorylated or low-abundance proteins were blotted first, and membranes were stripped using LI-COR NewBlot IR stripping buffer (LI-COR 928-40028; 30 m at a time until no signal remained from the first blot), then reblotted for non-phosphorylated or higher abundance proteins of similar sizes.

Images for both types of blots were background-subtracted and analyzed for band intensity with ImageLab software. Signals were normalized to a housekeeping protein quantified from the same gel (GAPDH, β-tubulin, β-actin). All original blots are available in Additional file 2.

Wherever possible, lysates were blotted on a ProteinSimple *WES* automated capillary-based size sorting system as previously described [30]. Briefly, lysates were mixed with reducing fluorescent master mix (ProteinSimple SM-001), heated (70 °C for 10 min) and loaded into manufacturer microplates containing primary antibodies (see below) and blocking reagent, wash buffer, HRP-conjugated secondary antibodies, and chemiluminescent substrate (ProteinSimple DM-001/2). *WES* data was analyzed on manufacturer-provided Compass software. All original data files presented in the style of a western blot membrane are provided in Additional file 2.

We used the following primary antibodies: NEEP21/NSG1 (Genscript A01442), FAM21 (Millipore ABT79), VPS35 (Abnova H00055737-M02), VPS26 (a kind gift from J. Bonifacino, NICHD), GluA1 (Millipore 05-855R), D2R (Millipore AB5084P), GluN1 (Millipore 05–432), LRRK2 (Abcam ab133474), LRRK2 phosphoS935 (Abcam ab133450), Rab10 phosphoT73 (Abcam ab230261), Rab10 (Abcam ab104859), VGluT1 (Millipore AB5905), β-tubulin (Covance MRB-435P), β-Actin (Abcam ab6276), and GAPDH (Cell Signaling 2118; ThermoFisher MA5-15738).

For co-immunoprecipitation, 500 µg of protein at 1 µg/µL was rotary-incubated overnight at 4 °C with VPS35 antibody (Abnova H00055737-M02) or Mouse IgG2a control antibody (Abcam ab18414) coupled to M-280 Tosyl-activated Dynabeads (Invitrogen 14204). Small aliquots of each lysate were set aside before IP to verify the equivalence of starting concentrations. 24 h later, loaded beads were washed with ice-cold lysis buffer (×3) prior to resuspension in reducing 1× NuPage LDS sample buffer (for traditional; Invitrogen NP0008) or ProteinSimple fluorescent master mix (for *WES*; ProteinSimple SM-001). Protein was eluted and denatured by heating at 70 °C for 10 min prior to western blotting as described above.

Antibody specificity was tested using lysate from LRRK2 knock-out mouse brain or Rab10 knock-out AtT20 cell lysates. The LRRK2 knock-out mice have been previously described by Hinkle and colleagues [81]. Rab10 knock-out AtT30 cells were a kind gift from Dr. Peter McPherson, and have been previously described [67].

### Primary cortical cultures

Dames were euthanized by rapid decapitation at embryonic day 16.5. Pups were microdissected in Hank’s Balanced Salt Solution (HBSS; Gibco 14170161) with 1 × penicillin–streptomycin (penstrep; Sigma-Aldrich P4333) in a petri dish on ice. The cortices from each pup were held in 500uL supplemented Hibernate-E medium (Gibco A1247601; 1 × GlutaMax Gibco 35050061; and 1% NeuroCult SM1 StemCell 5711) at 4 ℃ during genotyping as described above. Genotype-pooled tissue was dissociated chemically for 10 min in 0.05% Trypsin–EDTA (Gibco 25300054), followed by deactivation with 10% FBS, then mechanically dissociated in supplemented Neurobasal plating medium (Neurobasal Gibco 21103049; 1× GlutaMax Gibco 35050061; and 1% NeuroCult SM1 StemCell 5711).

Cells were plated onto poly-D-lysine-coated plates or coverslips (Sigma P7280) and matured to 21 days in-vitro (DIV21) while incubating at 37 ℃ with 5% CO_2_. For biochemistry, cells were plated at 1 million cells/well in 2 mL on 6-well plates. For immunocytochemistry experiments, cells were plated in 1 mL medium onto no.1.5 glass coverslips in 24-well plates. Non-nucleofected cells were plated at 115000 cells/well. For GFP-filled neurons, 1 million cells were nucleofected with 1 µg pAAV-CAG-GFP plasmid DNA (Addgene 37825) in Ingenio electroporation buffer (Mirus MIR50111) using an Amaxa Nucleofector2b (Lonza), mixed 1:1 with non-nucleofected cells, and plated at 225 k cells/well in 1 mL medium as above. From DIV4, 10% fresh media was added to all wells every 3–4 days until use.

### Immunocytochemistry and imaging analysis

Cultured cortical neurons were fixed at DIV21 (4% PFA, 4% sucrose in PBS, 5–10 min), permeabilized where appropriate (ice-cold methanol, 3 min), and blocked (5% goat serum in PBS) prior to incubation with primary antibodies for 1 h at RT or overnight at 4 ℃. Primary antibodies were prepared in antibody solution (2% goat serum, 0.02% Tween20 in PBS). For surface labelling, the cells were not permeabilized until after primary antibody incubation and no detergent was added to primary antibodies. Proteins were fluorescently labeled with Alexa-conjugated secondary (Invitrogen) in antibody solution for 30 min (RT), and coverslips were slide mounted with Prolong Gold (Invitrogen P36930).

We used the following primary antibodies: GFP (Abcam ab1218); VPS35 (Abnova H00055737); VPS26 (a kind gift from J. Bonifacino, NICHD); FAM21C (Millipore ABT79); NEEP21/NSG1 (Genscript A01442); Rab11 (Abcam ab95375); MAP2 (Abcam ab5392); GluA1 (Millipore 05-855R); PSD95 (Thermo Scientific MA1-045); VGluT1 (Millipore AB5905); GluA1 extracellular (Millipore ABN241); Rab10 (Abcam 237703); and GluA1 (Alomone AGP-009). Rab10 specificity was tested using Rab10 knock-out AtT30 cells (a kind gift from Dr. Peter McPherson; described in [67]) and cultured cortical neurons (Additional file 1: Fig. S8).

Cells having a pyramidal morphology—triangular or teardrop shaped cell bodies with spiny, clearly identifiable apical and basal dendrites [82]—were selected for imaging. All images were blinded and randomized prior to processing and analysis.

Cell density counts were performed manually on DAPI and MAP2 co-labeled images acquired at 10× with an Olympus Fluoview 1000 confocal microscope. Sholl analysis images were acquired at 20× with an Evos FL epifluorescence microscope. Dendritic neurites (excluding axons) were traced and analyzed using the Simple Neurite Tracer plugin for FIJI ImageJ using a radial segmentation of 5 µm.

Images for colocalization were acquired on either an Olympus Fluoview 1000 confocal microscope, with images taken at 60× with 2× optical zoom, in 0.5 µm stacks, or a Zeiss Axio Observer with Apotome.2 structured illumination upon which images were taken at 63× in 0.25 um stacks. Z-stack acquisition was set to capture all MAP2 stained dendrite for unfilled cells, or GFP-filled dendrite for filled cells. Acquisition parameters were constrained within each culture set. Z-stacks for each channel were flattened using the max projection function in FIJI. MAP2 or GFP stains were used to mask dendrites after their first branch point; primary dendrites and cell bodies were excluded from masks. Areas of the dendritic arbor with many intersecting neurites from other cells were excluded from analyses. Images were manually thresholded to create binary masks of clusters. Cluster densities, intensities, areas, colocalization densities, and Pearson’s coefficients were all calculated using automated pipelines in CellProfiler (www.cellprofiler.org; pipelines available upon request). Briefly, the pipeline first uses the dendrite mask to restrict all further analyses to the masked area. From there, the binary masks of clusters are used for the size, density, and colocalization densities within dendrites. Dendrite-masked greyscale images are used for Pearson’s coefficients, and greyscale images are overlaid with the cluster masks to measure intensity within clusters inside the dendritic region selected for analysis.

### In vitro electrophysiology

Whole-cell patch-clamp recordings were performed on cortical cells at DIV18-22. Neurons were perfused at room temperature with extra-cellular solution (167 mM NaCl, 2.4 mM KCl, 1 mM MgCl_2_, 10 mM glucose, 10 mM HEPES, 1 mM CaCl_2_, 1 µM tetrodotoxin and 100 µM picrotoxin, pH 7.4, 290–310 mOsm). In MLi-2 experiments, ECS was supplemented with 500 nM MLi-2 in 45% Captisol® PBS or an equal volume of 45% Captisol® PBS.

Pipettes were filled with intracellular solution (130 mM Cs-methanesulfonate, 5 mM CsCl, 4 mM NaCl, 1 mM MgCl_2_, 5 mM EGTA, 10 mM HEPES, 5 mM QX-314, 0.5 mM GTP, 10 mM Na_2_-phosphocreatine, 5 mM MgATP, and 0.1 mM spermine, at pH 7.3 and 290 mOsm). Pipette resistance was constrained to 3–7 MOhms for recording. Recordings were acquired by a Multiclamp 700B amplifier in voltage clamp mode at Vh -70 mV, signals were filtered at 2 kHz, digitized at 10 kHz. The membrane test function was used to determine intrinsic membrane properties 1–2 min after obtaining whole-cell configuration, as described previously [15, 83–85].

Tolerance for series resistance was < 28 мOhm and uncompensated, and recordings discarded if Rs changed by 10% or more. mEPSC frequency and amplitudes were analyzed with Clampfit 10 (Molecular Devices) with a detection threshold of 5pA and followed by manual confirmation of all accepted peaks; non-unitary events were suppressed from amplitude analysis but retained for frequency.

Peak-scaled nonstationary fluctuation analysis was performed on unitary events from each recording in the following way: events were aligned by peak amplitude, baselines adjusted, and all events normalized to −1 at their maximum amplitude. Event amplitudes and variance from the mean at each recording interval were calculated using the built-in NSFA plugin in Clampfit 10 (Molecular Devices), then rescaled to pre-normalization values. Mean–variance plots were made in GraphPad Prism using values from the event peak to 5pA above baseline (due to baseline noise), and fit using the least squares method and the second order polynomial function representing the following equation:

$$\delta^{2} = iI - \frac{{I^{2} }}{N} + \delta_{b}^{2}$$

where $\delta^{2}$ = variance, $i$ = single channel current, I = mean current, $\delta_{b}^{2}$ = background variance as previously described by others [86–89]. For conventional NSFA, $N$ = number of open channels at peak current; however the process of peak-normalizing required to analyze mEPSCs renders this value arbitrary. Weighted single-channel conductance was calculated by the following equation:

$$\gamma = i/(V_{m} - E_{rev)}$$

where $\gamma$ = weighted single-channel conductance, $i$ = single channel current, $V_{m}$ = holding potential (−70 mV), and $E_{rev}$ = reversal potential for AMPAR current (0 mV in our ECS). Recordings were rejected if the best-fit curve had an R^2^ < 0.5.

### LRRK2 kinase inhibition with MLi2 treatment

We inhibited LRRK2 kinase activity with the selective LRRK2 inhibitor MLi-2 (Tocris 5756). MLi-2 has low solubility in water, necessitating the use of a vehicle for solubilization. We chose to use the cyclodextrin Captisol® (Ligand RC-0C7-100) as a vehicle, due to its worldwide safety approval and use in human drug formulations (www.captisol.com/about). We bath sonicated 1 mg MLi-2 in 2 mL 45% Captisol®-PBS for ~ 2 h at room temperature (until complete solubilization of MLi-2). Solutions were filter sterilized prior to use. Primary cortical cultures were treated with 500 nM MLi-2 or Captisol®-only control (each 1 mL well treated with 0.4 uL stock in 100 mL fresh media; final Captisol® concentration 0.00016%) for 2 h prior to fixation, lysis, or electrophysiological recording. Treatment concentrations and times were selected based on the pharmacokinetic data collected by Fell and colleagues [50]. For western blot experiments in brain tissue, animals were injected intraperitoneally with MLi-2 or Captisol®-only control at a dose of 5 mg/kg, 2 h prior to rapid decapitation without anaesthesia. All tissues were collected, frozen, and stored as described above.

### Data visualization and statistics

All statistical analyses and data visualizations were conducted in GraphPad Prism 8. Because biological data is prone to lognormal distribution, outliers were only removed if inspection revealed that they resulted from human error. Data sets were analyzed for normality using the D’Agostino & Pearson test. In untreated experiments and drug effect comparisons (3 groups) when the data failed the normality test (alpha < 0.05), nonparametric tests were used (Kruskal–Wallis with uncorrected Dunn’s post-test). If the data passed the normality test, (alpha > 0.05), a parametric test was chosen. When the SDs were not significantly different, a one-way analysis of variance (ANOVA) with uncorrected Fisher’s LSD post-test was used. If the SDs were significantly different, Welch’s ANOVA and an unpaired t-test with Welch’s correction post-test was used. In the event that the n was too low for normality testing, nonparametric tests were used. For Captisol® tests (2 groups), the tests chosen were appropriate for two groups (Mann–Whitney for nonparametric and unpaired two-tailed t-test for parametric). MLi-2 treatment data was analyzed using 2-way ANOVA (2-way ANOVA) with uncorrected Fisher’s LSD post-tests.

Sample sizes were selected according to generally accepted standards in the field. No power analyses were conducted to predetermine sample sizes. Data is represented as scatter plots of all data points with mean and standard error of the mean. Sample sizes represent biological replicates. Where n = x(y), x = number of cells imaged/recorded, y = number of independent cultures.

## Supplementary Information

**Additional file 1: Fig. S1.** Retromer protein levels not altered in VKI. **A**) Co-immunoprecipitation performed in VKI whole brain lysate pulling with FAM21 antibody and blotted on a WES capillary-based western blotting system shows VPS35 association with FAM21 in brain tissue. **B**) WES capillary-based western blot of VPS35, VPS26, FAM21, and β-tubulin in VKI whole-brain lysate (i) revealed no significant genotype effects on levels of VPS35 (ii, 1-way ANOVA p = 0.97), VPS26 (iii, Kruskal–Wallis p = 0.73), or WASH complex member FAM21 (iv, 1-way ANOVA p = 0.88). **Fig. S2.** Neuronal cargo and LRRK2 binding are not altered i n VKI. A) Western blot of striatal lysates and co-immunoprecipitates from 3-month-old VKI mice (pulling with VPS35 antibody) were probed for VPS35, GluN1, D2R, GluA1, LRRK2, and GAPDH (i). There were no genotype effects on VPS35 levels or pull by the antibody (ii–iii, Kruskal–Wallis p = 0.97; p = 0.13, respectively); GluN1 levels or coIP (iv–v, Kruskal–Wallis p = 0.51; p = 0.42, respectively); D2R levels or coIP (vi–vii Kruskal–Wallis p = 0.70; p = 0.45, respectively); GluA1 levels or coIP (viii–ix, Kruskal–Wallis p = 0.83; p = 0.44, respectively); or LRRK2 levels or coIP (x–xi, Kruskal–Wallis p > 0.99; p = 0.40, respectively). **Fig. S3.** Phospho-LRRK2 is increased in VKI and MLi-2 does not alter protein levels. **A)** Western blot of LRRK2 pS935, LRRK2, and β-actin (i) revealed no genotype effect on LRRK2 expression levels (ii, Kruskal–Wallis p = 0.09). LRRK2 pS935 levels were significantly altered, due to an increase in phosphorylation in homozygous tissue (iii, 1-way ANOVA p < 0.03; Uncorrected Fisher’s LSD *p < 0.02). **B.i**) Whole brain lysates from VKI animals after acute LRRK2 kinase inhibition were blotted for LRRK2, GluA1, VPS35, VGluT1, Rab10, and β-actin (loading control). There were no significant effects of genotype or treatment on levels of LRRK2 (ii, 2-way ANOVA genotype x treatment p = 0.93; genotype p = 0.90; treatment p = 0.24), GluA1 (iii, 2-way ANOVA interaction p = 0.45; genotype p = 0.61; treatment p > 0.99), VPS35 (vi, 2-way ANOVA interaction p = 0.23; genotype p = 0.94; treatment p = 0.89), VGluT1 (vii, 2-way ANOVA interaction p = 0.55; genotype p = 0.39; treatment p = 0.69), or Rab10 (iv, 2-way ANOVA genotype x treatment p = 0.5258; genotype p = 0.4683; treatment p = 0.9659). For Bii-vi, WTCap n = 5, WTMLi2 n = 6, HetCap n = 6, HetMLi2 n = 6, HoCap n = 5, HoMLi2 n = 5. **Fig. S4.** Captisol does not affect protein levels**. A**) Further analysis of blots from Fig. 1. There were no significant genotype effects on the level of LRRK2 (i; Mann–Whitney p > 0.99) or Rab10 (ii; Mann–Whitney p = 0.89). **B**) Western blots of WT brain lysate following acute treatment with Captisol or saline, probed for GluA1, VPS35, VGluT1, and β-actin (loading control). There were no significant effects of Captisol treatment on protein levels of VPS35, GluA1, or VGluT1 (ii–iv; Mann–Whitney p = 0.49; p > 0.99; p = 0.49, respectively). **Fig. S5.** Cell density and dendritic morphology are not altered in VKI. **A**) Cortical cells were nucleofected with CAG-AAV-GFP plasmids on the day of plating and fixed at DIV21. GFP signal was amplified and imaged (top panel), then 2D in silico cell reconstruction performed in ImageJ (bottom panel). **B**) There was no effect of genotype on neuron density, indicating equivalent survival and no cell death (1-way ANOVA p = 0.47). **C**) Sholl analysis revealed no significant effect of genotype upon neurite complexity (2-way RM ANOVA radial distance x genotype interaction p = 0.87; genotype p = 0.78). **D-F**) There were also no genotype effects on total branch number, average branch length, or maximum branch length (Kruskal–Wallis p = 0.79, 0.34 & 0.29, respectively). **Fig. S6.** VGluT1 cluster intensity reduced in VKI and channel kinetics not affected. **A**) Supplemental analysis from untreated cortical cell culture synapse staining presented in Fig. 3. PSD95 and VGluT1 densities were not altered by genotype (i-ii, Welch’s ANOVA p = 0.29 & 0.42, respectively). There was a genotype effect on VGluT1 cluster intensity, due to significant reductions in homozygous cells only (iii, Kruskal–Wallis p < 0.007; Uncorrected Dunn’s **p < 0.003). **B**) Supplemental analysis of whole-cell patch clamp recordings from cultured cortical cells presented in Fig. 3. Mean mEPSC decay times (τ) were not affected by genotype (i, Kruskal–Wallis p = 0.74). Peak-scaled non-stationary noise analysis was performed by plotting the mean variance of traces from the recording average amplitude (ii, representative mean–variance plots); the best fit curve allows for the calculation of weighted single channel conductance of the synapses involved in each recording. Calculation of weighted single channel conductance from best-fit curves revealed no genotype effect on single channel conductance (iii, Kruskal Wallis p = 0.78). **Fig. S7.** LRRK2 is expressed and phosphorylated in cultured neurons, and MLi-2 decreases pLRRK2. Fluorscence western blot of pLRRK2, LRRK2, and GAPDH VKI cortical culture lysate revealed that the presence of LRRK2 and LRRK2 p935 in vehicle treated cultures, and absence of pLRRK2 following acute MLi-2 treatment; however, due to low stoichiometry bands were not high enough above background to be reliably quantified. **Fig. S8.** GluA1 levels unaltered but dendritic cluster density is reduced in VKI. A) Western blot of GluA1 and β-actin in cortical lysates of VKI mice (i) revealed no genotype effect on GluA1 protein levels (ii, Kruskal–Wallis p = 0.99). **B**) Co-immunoprecipitation of GluA1 with VPS35 (i) revealed no genotype effect (ii, Kruskal–Wallis p = 0.99). **C**) Cultured cortical neurons immunostained for MAP2 (blue), VPS35 (cyan), and GluA1 (magenta) (i). There was a significant reduction in GluA1 cluster density in homozygous VKI neurons (ii, Kruskal–Wallis p < 0.02; Uncorrected Dunn’s **p < 0.005) and no genotype effect on VPS35-GluA1 co-cluster density of (iii, 1-way ANOVA p = 0.13), or Pearson’s coefficient (iv, Kruskal–Wallis p = 0.57). **Fig. S9.** Rab10 does not colocalize strongly with GluA1 in cortical neurites. **A**) GFP-filled (blue) cortical neurons immunostained for Rab10 (magenta), and GluA1 (cyan) (i). Rab10 cluster density was increased in both mutant genotypes, falling just shy of statistical significance (ii, Kruskal–Wallis p < 0.06). There was no effect of genotype on co-cluster density or Pearson’s coefficient (iii–iv, 1-way ANOVA p = 0.24; Kruskal–Wallis p = 0.56, respectively). **B**) Knock-out testing of specificity of Rab10 antibody for immunocytochemistry. Cultured cortical neurons and Rab10 knock-out AtT30 cells were stained by immunocytochemistry for Rab10, demonstrating punctate staining in the cortical neuron that is absent from the knock-out cells.

**Additional file 2:** Western blot images. Original western blot images.

## Data Availability

All data generated or analyzed during this study are included in this published article (and its Additional files 1, 2).

## Abbreviations

AMPA: Alpha-amino-3-hydroxy-5-methyl-4-isoxazolepropionic acid
AMPAR: Alpha-amino-3-hydroxy-5-methyl-4-isoxazolepropionic acid receptor
Captisol®: Sulfobutylether-β-cyclodextrin
CI-MPR: Cation-independent mannose-6-phosphate receptor
coIP: Co-immunoprecipitation
D2R: D2-type dopamine receptor
DAT: Dopamine transporter
FAM21: Family with sequence similarity 21
LRRK2: Leucine-rich repeat kinase 2
LTP: Long-term potentiation
mEPSC: Miniature excitatory post-synaptic current
NEEP21: Neuronal endosomal enriched protein 21
NMDA: N-methyl-D-aspartate
NSFA: Non-stationary fluctuation analysis
PD: Parkinson’s disease
PKA: Protein kinase A
Pr: Probability of release
PSD95: Post-synaptic density protein 95
RE: Recycling endosome
SV: Synaptic vesicle
TGN: *trans*-Golgi network
VGluT1: Vesicular glutamate transporter 1
VKI: VPS35 D620N knock-in
VMAT2: Vesicular monoamine transporter 2
VPS26: Vacuolar protein sorting 26
VPS35: Vacuolar protein sorting 35
WASH: Wiskott–Aldrich syndrome protein and SCAR homolog

## References

[1] Burd C, Cullen PJ (2014). Retromer: a master conductor of endosome sorting. Cold Spring Harb Perspect Biol..

[2] Bonifacino JS, Rojas R (2006). Retrograde transport from endosomes to the trans-Golgi network. Nat Rev Mol Cell Biol..

[3] Cullen PJ, Korswagen HC (2011). Sorting nexins provide diversity for retromer-dependent trafficking events. Nat Cell Biol..

[4] Seaman MNJ, Gautreau A, Billadeau DD (2013). Retromer-mediated endosomal protein sorting: all WASHed up!. Trends Cell Biol..

[5] Struhal W, Presslauer S, Spielberger S, Zimprich A, Auff E, Bruecke T (2014). VPS35 Parkinson’s disease phenotype resembles the sporadic disease. J Neural Transm..

[6] Vilariño-Güell C, Wider C, Ross OA, Dachsel JC, Kachergus JM, Lincoln SJ (2011). VPS35 mutations in Parkinson disease. Am J Hum Genet..

[7] Zimprich A, Benet-Pagès A, Struhal W, Graf E, Eck SH, Offman MN (2011). A mutation in VPS35, encoding a subunit of the retromer complex, causes late-onset Parkinson disease. Am J Hum Genet..

[8] Kordower JH, Olanow CW, Dodiya HB, Chu Y, Beach TG, Adler CH (2013). Disease duration and the integrity of the nigrostriatal system in Parkinson’s disease. Brain..

[9] Beitz JM (2014). Parkinson’s disease: a review. Front Biosci.

[10] Goldman JG, Postuma R (2014). Premotor and nonmotor features of Parkinson’s disease. Curr Opin Neurol..

[11] Foffani G, Obeso JA (2018). A cortical pathogenic theory of Parkinson’s disease. Neuron.

[12] Chen X, Kordich JK, Williams ET, Levine N, Cole-Strauss A, Marshall L (2019). Parkinson’s disease-linked D620N VPS35 knock-in mice manifest tau neuropathology and dopaminergic neurodegeneration. Proc Natl Acad Sci U S A.

[13] Niu M, Zhao F, Bondelid K, Siedlak SL, Torres S, Fujioka H (2021). VPS35 D620N knockin mice recapitulate cardinal features of Parkinson’s disease. Aging Cell.

[14] Choy RW-Y, Park M, Temkin P, Herring BE, Marley A, Nicoll RA (2014). Retromer mediates a discrete route of local membrane delivery to dendrites. Neuron..

[15] Munsie L, Milnerwood A, Seibler P, Beccano-Kelly D, Tatarnikov I, Khinda J (2015). Retromer-dependent neurotransmitter receptor trafficking to synapses is altered by the Parkinson’s disease VPS35 mutation p.D620N. Hum Mol Genet..

[16] Tian Y, Tang F-L, Sun X, Wen L, Mei L, Tang B-S (2015). VPS35-deficiency results in an impaired AMPA receptor trafficking and decreased dendritic spine maturation. Mol Brain..

[17] Tsika E, Glauser L, Moser R, Fiser A, Daniel G, Sheerin U-M (2014). Parkinson’s disease-linked mutations in VPS35 induce dopaminergic neurodegeneration. Hum Mol Genet..

[18] Zhang D, Isack NR, Glodowski DR, Liu J, Chen CCH, Xu XZS (2012). RAB-62 and the retromer regulate glutamate receptor recycling through a retrograde pathway. J Cell Biol..

[19] Wang C-L, Tang F-L, Peng Y, Shen C-Y, Mei L, Xiong W-C (2012). VPS35 regulates developing mouse hippocampal neuronal morphogenesis by promoting retrograde trafficking of BACE1. Biol Open..

[20] Tang FL, Zhao L, Zhao Y, Sun D, Zhu XJ, Mei L, et al. Coupling of terminal differentiation deficit with neurodegenerative pathology in Vps35-deficient pyramidal neurons. Cell Death Differ. 2020;7:2099–116. 10.1038/s41418-019-0487-2 PMC7308361 31907392

[21] Temkin P, Morishita W, Goswami D, Arendt K, Chen L, Malenka R (2017). The retromer supports AMPA receptor trafficking during LTP. Neuron..

[22] Vazquez-Sanchez S, Bobeldijk S, Dekker MP, Van Keimpema L, Van Weering JRT (2018). VPS35 depletion does not impair presynaptic structure and function. Sci Rep..

[23] Linhart R, Wong SA, Cao J, Tran M, Huynh A, Ardrey C (2014). Vacuolar protein sorting 35 (Vps35) rescues locomotor deficits and shortened lifespan in Drosophila expressing a Parkinson’s disease mutant of Leucine-Rich Repeat Kinase 2 (LRRK2). Mol Neurodegener..

[24] MacLeod DA, Rhinn H, Kuwahara T, Zolin A, Di Paolo G, McCabe BD (2013). RAB7L1 interacts with LRRK2 to modify intraneuronal protein sorting and Parkinson’s disease risk. Neuron.

[25] Inoshita T, Arano T, Hosaka Y, Meng H, Umezaki Y, Kosugi S (2017). Vps35 in cooperation with LRRK2 regulates synaptic vesicle endocytosis through the endosomal pathway in Drosophila. Hum Mol Genet.

[26] Zhao Y, Perera G, Takahashi-Fujigasaki J, Mash DC, Vonsattel JPG, Uchino A (2018). Reduced LRRK2 in association with retromer dysfunction in post-mortem brain tissue from LRRK2 mutation carriers. Brain.

[27] Mir R, Tonelli F, Lis P, Macartney T, Polinski NK, Martinez TN (2018). The Parkinson’s disease VPS35[D620N] mutation enhances LRRK2-mediated Rab protein phosphorylation in mouse and human. Biochem J.

[28] Vilariño-Güell C, Rajput A, Milnerwood AJ, Shah B, Szu-Tu C, Trinh J, et al. DNAJC13 mutations in Parkinson disease. Hum Mol Genet. 2013;1–8. 10.1093/hmg/ddt570 PMC3999380 24218364

[29] Healy DG, Falchi M, O’Sullivan SS, Bonifati V, Durr A, Bressman S (2008). Phenotype, genotype, and worldwide genetic penetrance of LRRK2-associated Parkinson’s disease: a case-control study. Lancet Neurol..

[30] Beccano-Kelly DA, Kuhlmann N, Tatarnikov I, Volta M, Munsie LN, Chou P (2014). Synaptic function is modulated by LRRK2 and glutamate release is increased in cortical neurons of G2019S LRRK2 knock-in mice. Front Cell Neurosci..

[31] Beccano-Kelly DA, Volta M, Munsie LN, Paschall SA, Tatarnikov I, Co K (2014). LRRK2 overexpression alters glutamatergic presynaptic plasticity, striatal dopamine tone, postsynaptic signal transduction, motor activity and memory. Hum Mol Genet..

[32] Cirnaru MD, Marte A, Belluzzi E, Russo I, Gabrielli M, Longo F (2014). LRRK2 kinase activity regulates synaptic vesicle trafficking and neurotransmitter release through modulation of LRRK2 macro-molecular complex. Front Mol Neurosci..

[33] Piccoli G, Condliffe SB, Bauer M, Giesert F, Boldt K, De Astis S (2011). LRRK2 controls synaptic vesicle storage and mobilization within the recycling pool. J Neurosci..

[34] Volta M, Cataldi S, Beccano-Kelly D, Munsie L, Tatarnikov I, Chou P (2015). Chronic and acute LRRK2 silencing has no long-term behavioral effects, whereas wild-type and mutant LRRK2 overexpression induce motor and cognitive deficits and altered regulation of dopamine release. Parkinsonism Relat Disord..

[35] Matikainen-Ankney BA, Kezunovic N, Mesias RE, Tian Y, Williams FM, Huntley GW (2016). Altered development of synapse structure and function in striatum caused by Parkinson’s disease-linked LRRK2-G2019S mutation. J Neurosci..

[36] Parisiadou L, Yu J, Sgobio C, Xie C, Liu G, Sun L (2014). LRRK2 regulates synaptogenesis and dopamine receptor activation through modulation of PKA activity. Nat Neurosci.

[37] Sweet ES, Saunier-Rebori B, Yue Z, Blitzer RD (2015). The Parkinson’s disease-associated mutation LRRK2-G2019S impairs synaptic plasticity in mouse hippocampus. J Neurosci.

[38] Matikainen-Ankney BA, Kezunovic N, Menard C, Flanigan ME, Zhong Y, Russo SJ (2018). Parkinson’s disease-linked lrrk2-g2019s mutation alters synaptic plasticity and promotes resilience to chronic social stress in young adulthood. J Neurosci.

[39] Taylor M, Alessi DR (2020). Advances in elucidating the function of leucine-rich repeat protein kinase-2 in normal cells and Parkinson’s disease. Curr Opin Cell Biol..

[40] Kuhlmann N, Milnerwood AJ (2020). A critical LRRK at the synapse? The neurobiological function and pathophysiological dysfunction of LRRK2. Front Mol Neurosci.

[41] Steger M, Tonelli F, Ito G, Davies P, Trost M, Vetter M, et al. Phosphoproteomics reveals that Parkinson’s disease kinase LRRK2 regulates a subset of Rab GTPases. Elife. 2016. 10.7554/eLife.12813. 10.7554/eLife.12813 PMC4769169 26824392

[42] Jeong GR, Jang EH, Bae JR, Jun S, Kang HC, Park CH (2018). Dysregulated phosphorylation of Rab GTPases by LRRK2 induces neurodegeneration. Mol Neurodegener.

[43] Di Maio R, Hoffman EK, Rocha EM, Keeney MT, Sanders LH, De Miranda BR (2018). LRRK2 activation in idiopathic Parkinson’s disease. Sci Transl Med..

[44] McGough IJ, Steinberg F, Jia D, Barbuti PA, McMillan KJ, Heesom KJ (2014). Retromer binding to FAM21 and the WASH complex is perturbed by the parkinson disease-linked VPS35(D620N) mutation. Curr Biol..

[45] Zavodszky E, Seaman MNJ, Moreau K, Jimenez-Sanchez M, Breusegem SY, Harbour ME (2014). Mutation in VPS35 associated with Parkinson’s disease impairs WASH complex association and inhibits autophagy. Nat Commun..

[46] Follett J, Norwood SJ, Hamilton NA, Mohan M, Kovtun O, Tay S, et al. The Vps35 D620N mutation linked to Parkinson’s disease disrupts the cargo sorting function of retromer. Traffic. 2013;(6). 10.1111/tra.12136 24152121

[47] Cui Y, Yang Z, Flores-Rodriguez N, Follett J, Ariotti N, Wall AA (2021). Formation of retromer transport carriers is disrupted by the Parkinson disease-linked Vps35 D620N variant. Traffic.

[48] Cataldi S, Follett J, Fox JD, Tatarnikov I, Kadgien C, Gustavsson EK (2018). Altered dopamine release and monoamine transporters in Vps35 pD620N knock-in mice. npj Park Dis..

[49] Kluss JH, Mazza MC, Li Y, Manzoni C, Lewis PA, Cookson MR (2021). Preclinical modeling of chronic inhibition of the Parkinson’s disease associated kinase LRRK2 reveals altered function of the endolysosomal system in vivo. Mol Neurodegener.

[50] Fell MJ, Mirescu C, Basu K, Cheewatrakoolpong B, DeMong DE, Ellis JM (2015). MLi-2, a potent, selective, and centrally active compound for exploring the therapeutic potential and safety of LRRK2 kinase inhibition. J Pharmacol Exp Ther.

[51] Gomez TS, Billadeau DD (2009). A FAM21-containing WASH complex regulates retromer-dependent sorting. Dev Cell..

[52] Derivery E, Sousa C, Gautier JJ, Lombard B, Loew D, Gautreau A (2009). The Arp2/3 activator WASH controls the fission of endosomes through a large multiprotein complex. Dev Cell..

[53] Lee S, Chang J, Blackstone C (2016). FAM21 directs SNX27-retromer cargoes to the plasma membrane by preventing transport to the Golgi apparatus. Nat Commun..

[54] Yap CC, Digilio L, McMahon L, Winckler B (2017). The endosomal neuronal proteins Nsg1/NEEP21 and Nsg2/P19 are itinerant, not resident proteins of dendritic endosomes. Nat Sci Rep..

[55] Bodrikov V, Pauschert A, Kochlamazashvili G, Stuermer CAO (2017). Reggie-1 and reggie-2 (flotillins) participate in Rab11a-dependent cargo trafficking, spine synapse formation and LTP-related AMPA receptor (GluA1) surface exposure in mouse hippocampal neurons. Exp Neurol..

[56] Correia SS, Bassani S, Brown TC, Lisé M-F, Backos DS, El-Husseini A (2008). Motor protein-dependent transport of AMPA receptors into spines during long-term potentiation. Nat Neurosci..

[57] da Silva EM, Adrian M, Schätzle P, Lipka J, Watanabe T, Cho S (2015). Positioning of AMPA receptor-containing endosomes regulates synapse architecture. Cell Rep..

[58] Jaafari N, Henley JM, Hanley JG (2012). PICK1 mediates transient synaptic expression of GluA2-lacking AMPA receptors during glycine-induced AMPA receptor trafficking. J Neurosci.

[59] Seebohm G, Neumann S, Theiss C, Novkovic T, Hill EV, Tavaré JM, et al. Identification of a novel signaling pathway and its relevance for GluA1 recycling. PLoS One. 2012;7(3):e33889. 10.1371/journal.pone.0033889 PMC3309939 22470488

[60] Bowen AB, Bourke AM, Hiester BG, Hanus C, Kennedy MJ (2017). Golgi-Independent secretory trafficking through recycling endosomes in neuronal dendrites and spines. Elife.

[61] Benke T, Traynelis SF (2019). AMPA-type glutamate receptor conductance changes and plasticity: still a lot of noise. Neurochem Res.

[62] Diering GH, Huganir RL (2018). The AMPA receptor code of synaptic plasticity. Neuron..

[63] Wang P, Liu H, Wang Y, Liu O, Zhang J, Gleason A (2016). RAB-10 promotes EHBP-1 bridging of filamentous actin and tubular recycling endosomes. PLoS Genet.

[64] Bruno J, Brumfield A, Chaudhary N, Iaea D, McGraw TE (2016). SEC16A is a RAB10 effector required for insulinstimulated GLUT4 trafficking in adipocytes. J Cell Biol.

[65] Chen Y, Wang Y, Zhang J, Deng Y, Jiang L, Song E (2012). Rab10 and myosin-va mediate insulin-stimulated GLUT4 storage vesicle translocation in adipocytes. J Cell Biol.

[66] Pan X, Zaarur N, Singh M, Morin P, Kandror KV (2017). Sortilin and retromer mediate retrograde transport of Glut4 in 3T3-L1 adipocytes. Mol Biol Cell.

[67] Alshafie W, Francis V, Bednarz K, Pan YE, Stroh T, McPherson PS. Regulated resurfacing of a somatostatin receptor storage compartment fine-tunes pituitary secretion. J Cell Biol. 2020. 10.1083/jcb.201904054. 10.1083/jcb.201904054 PMC7039187 31825461

[68] Glodowski DR, Chen CC-H, Schaefer H, Grant BD, Rongo C (2007). RAB-10 regulates glutamate receptor recycling in a cholesterol-dependent endocytosis pathway. Mol Biol Cell..

[69] Pavlos NJ, Grønborg M, Riedel D, Chua JJE, Boyken J, Kloepper TH (2010). Quantitative analysis of synaptic vesicle Rabs uncovers distinct yet overlapping roles for Rab3a and Rab27b in Ca2+-triggered exocytosis. J Neurosci.

[70] Tang F-L, Erion JR, Tian Y, Liu W, Yin D-M, Ye J (2015). VPS35 in dopamine neurons is required for endosome-to-golgi retrieval of Lamp2a, a receptor of chaperone-mediated autophagy that is critical for -synuclein degradation and prevention of pathogenesis of Parkinson’s disease. J Neurosci..

[71] Wen L, Tang F, Hong Y, Luo S, Wang C, He W (2011). VPS35 haploinsufficiency increases Alzheimer’s disease neuropathology. J Cell Biol.

[72] Seaman MNJ (2018). Retromer and the cation-independent mannose 6-phosphate receptor—time for a trial separation?. Traffic.

[73] Wang C, Niu M, Zhou Z, Zheng X, Zhang L, Tian Y (2016). VPS35 regulates cell surface recycling and signaling of dopamine receptor D1. Neurobiol Aging..

[74] Volta M, Beccano-Kelly DA, Paschall SA, Cataldi S, Macisaac SE, Kuhlmann N, et al. Initial elevations in glutamate and dopamine neurotransmission decline with age, as does exploratory behavior, in LRRK2 G2019S knock-in mice. Elife. 2017;6. 10.7554/eLife.28377 PMC5633343 28930069

[75] Yue M, Hinkle K, Davies P, Trushina E, Fiesel F, Christenson T (2015). Progressive dopaminergic alterations and mitochondrial abnormalities in LRRK2 G2019S knock in mice. Neurobiol Dis..

[76] West AB, Moore DJ, Biskup S, Bugayenko A, Smith WW, Ross CA (2005). Parkinson’s disease-associated mutations in leucine-rich repeat kinase 2 augment kinase activity. Proc Natl Acad Sci U S A.

[77] Steger M, Diez F, Dhekne HS, Lis P, Nirujogi RS, Karayel O (2017). Systematic proteomic analysis of LRRK2-mediated rab GTPase phosphorylation establishes a connection to ciliogenesis. Elife..

[78] Schreij AM, Chaineau M, Ruan W, Lin S, Barker PA, Fon EA (2015). LRRK2 localizes to endosomes and interacts with clathrin-light chains to limit Rac1 activation. EMBO Rep..

[79] Picconi B, Piccoli G, Calabresi P. Synaptic Dysfunction in Parkinson’s Disease. In: Kreutz MR, Sala C, editors. Vienna: Springer Vienna; 2012 (cited 2013 Dec 12). Advances in Experimental Medicine and Biology; vol. 970; p. 553–72. 10.1007/978-3-7091-0932-8. 10.1007/978-3-7091-0932-8_24 22351072

[80] Volta M, Milnerwood AJ, Farrer MJ (2015). Insights from late-onset familial parkinsonism on the pathogenesis of idiopathic Parkinson’s disease. Lancet Neurol..

[81] Hinkle KM, Yue M, Behrouz B, Dächsel JC, Lincoln SJ, Bowles EE (2012). LRRK2 knockout mice have an intact dopaminergic system but display alterations in exploratory and motor co-ordination behaviors. Mol Neurodegener.

[82] Kriegstein AR, Dichter MA (1983). Morphological classification of rat cortical neurons in cell culture. J Neurosci.

[83] Kaufman AM, Milnerwood AJ, Sepers MD, Coquinco A, She K, Wang L (2012). Opposing roles of synaptic and extrasynaptic NMDA receptor signaling in cocultured striatal and cortical neurons. J Neurosci..

[84] Brigidi GS, Sun Y, Beccano-Kelly D, Pitman K, Mobasser M, Borgland SL (2014). Palmitoylation of δ-catenin by DHHC5 mediates activity-induced synapse plasticity. Nat Neurosci..

[85] Milnerwood AJ, Kaufman AM, Sepers MD, Gladding CM, Zhang L, Wang L (2012). Mitigation of augmented extrasynaptic NMDAR signaling and apoptosis in cortico-striatal co-cultures from Huntington’s disease mice. Neurobiol Dis..

[86] Traynelis SF, Silver RA, Cull-Candy SC (1993). Estimated conductance of G lutamate Receptor Channels Activated during EPSCs at the Cerebellar Mossy Fiber-Granule Cell Synapse. Neuron.

[87] Hartveit E, Veruki ML (2007). Studying properties of neurotransmitter receptors by non-stationary noise analysis of spontaneous postsynaptic currents and agonist-evoked responses in outside-out patches. Nat Protoc.

[88] Smith-Dijak AI, Nassrallah WB, Zhang LYJ, Geva M, Hayden MR, Raymond LA (2019). Impairment and restoration of homeostatic plasticity in cultured cortical neurons from a mouse model of huntington disease. Front Cell Neurosci..

[89] Benke TA, Lüthi A, Palmer MJ, Wikström MA, Anderson WW, Isaac JTR (2001). Mathematical modelling of non-stationary fluctuation analysis for studying channel properties of synaptic AMPA receptors. J Physiol.

