# Supplementary material for: Endosomal traffic and glutamate synapse activity are increased in VPS35 D620N mutant knock-in mouse neurons, and resistant to LRRK2 kinase inhibition
Source: Mol Brain. 2021 Sep 16;14:143. doi: 10.1186/s13041-021-00848-w (PMC8447518; doi:10.1186/s13041-021-00848-w)
Supplement: Supplementary file 1 — Additional file 1: Fig. S1. Retromer protein levels not altered in VKI. A) Co-immunoprecipitation performed in VKI whole brain lysate pulling with FAM21 antibody and blotted on a WES capillary-based western blotting system shows VPS35 association with FAM21 in brain tissue. B) WES capillary-based western blot of VPS35, VPS26, FAM21, and β-tubulin in VKI whole-brain lysate (i) revealed no significant genotype effects on levels of VPS35 (ii, 1-way ANOVA p = 0.97), VPS26 (iii, Kruskal–Wallis p = 0.73), or WASH complex member FAM21 (iv, 1-way ANOVA p = 0.88). Fig. S2. Neuronal cargo and LRRK2 binding are not altered i n VKI. A) Western blot of striatal lysates and co-immunoprecipitates from 3-month-old VKI mice (pulling with VPS35 antibody) were probed for VPS35, GluN1, D2R, GluA1, LRRK2, and GAPDH (i). There were no genotype effects on VPS35 levels or pull by the antibody (ii–iii, Kruskal–Wallis p = 0.97; p = 0.13, respectively); GluN1 levels or coIP (iv–v, Kruskal–Wallis p = 0.51; p = 0.42, respectively); D2R levels or coIP (vi–vii Kruskal–Wallis p = 0.70; p = 0.45, respectively); GluA1 levels or coIP (viii–ix, Kruskal–Wallis p = 0.83; p = 0.44, respectively); or LRRK2 levels or coIP (x–xi, Kruskal–Wallis p > 0.99; p = 0.40, respectively). Fig. S3. Phospho-LRRK2 is increased in VKI and MLi-2 does not alter protein levels. A) Western blot of LRRK2 pS935, LRRK2, and β-actin (i) revealed no genotype effect on LRRK2 expression levels (ii, Kruskal–Wallis p = 0.09). LRRK2 pS935 levels were significantly altered, due to an increase in phosphorylation in homozygous tissue (iii, 1-way ANOVA p < 0.03; Uncorrected Fisher’s LSD *p < 0.02). B.i) Whole brain lysates from VKI animals after acute LRRK2 kinase inhibition were blotted for LRRK2, GluA1, VPS35, VGluT1, Rab10, and β-actin (loading control). There were no significant effects of genotype or treatment on levels of LRRK2 (ii, 2-way ANOVA genotype x treatment p = 0.93; genotype p = 0.90; treatment p = 0.24), GluA1 (iii, 2-way [file 13041_2021_848_MOESM1_ESM.pdf]

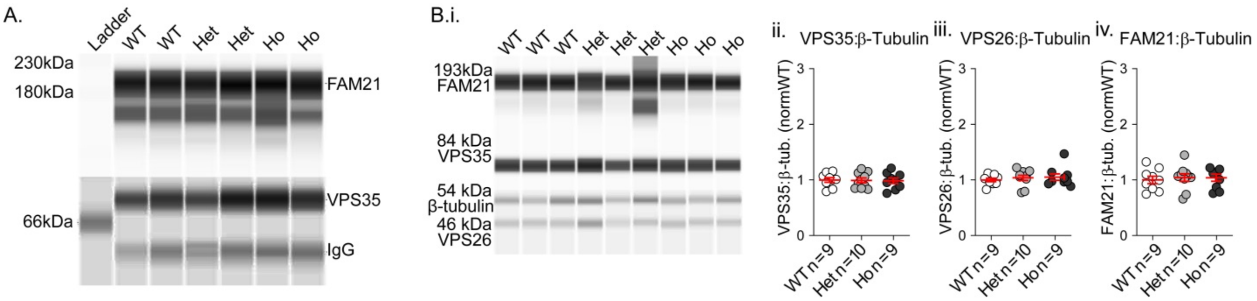


**Additional file 1: Figure S1 Retromer protein levels not altered in VKI. A)** Co-immunoprecipitation performed in VKI whole brain lysate pulling with FAM21 antibody and blotted on a WES capillary-based western blotting system shows VPS35 association with FAM21 in brain tissue. **B)** WES capillary-based western blot of VPS35, VPS26 , FAM21, and β-tubulin in VKI whole-brain lysate (i) revealed no significant genotype effects on levels of VPS35 (ii, 1-way ANOVA $p$=0.97), VPS26 (iii, Kruskal-Wallis $p$=0.73), or WASH complex member FAM21 (iv, 1-way ANOVA $p$=0.88).

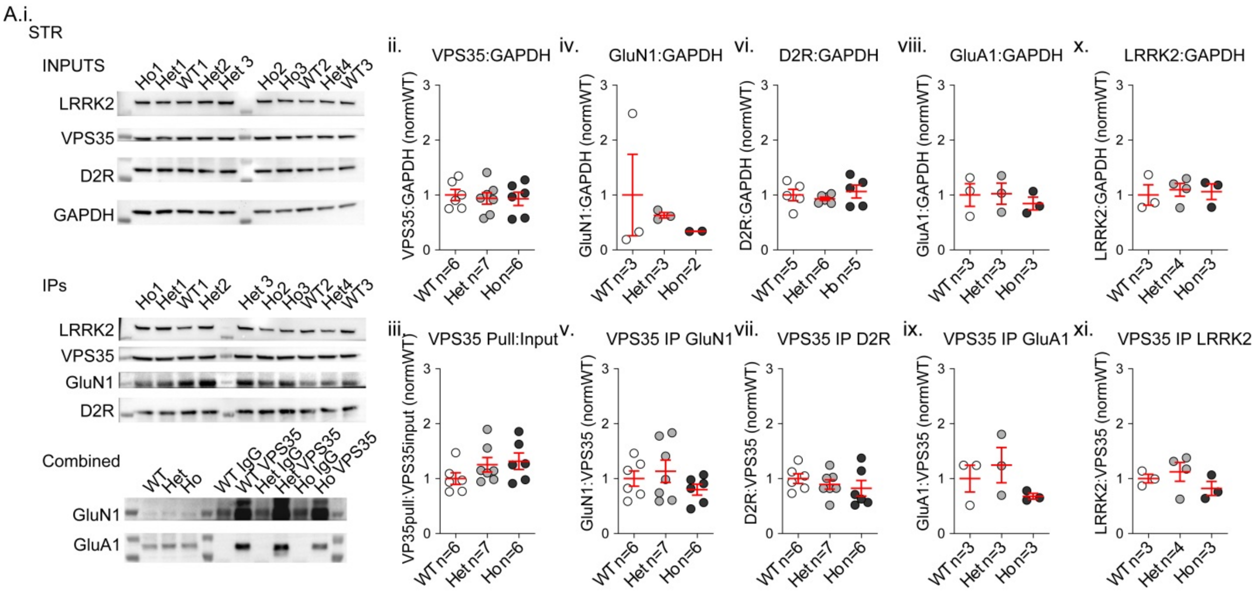


**Additional file 1: Figure S2 Neuronal cargo and LRRK2 binding are not altered in VKI.** A) Western blot of striatal lysates and co-immunoprecipitates from 3-month-old VKI mice (pulling with VPS35 antibody) were probed for VPS35, GluN1, D2R, GluA1, LRRK2, and GAPDH (i). There were no genotype effects on VPS35 levels or pull by the antibody (ii-iii, Kruskal-Wallis $p$=0.97; $p$=0.13, respectively); GluN1 levels or coIP (iv-v, Kruskal-Wallis $p$=0.51; $p$=0.42, respectively); D2R levels or coIP (vi-vii Kruskal-Wallis $p$=0.70; $p$=0.45, respectively); GluA1 levels or coIP (viii-ix, Kruskal-Wallis $p$=0.83; $p$=0.44, respectively); or LRRK2 levels or coIP (x-xi, Kruskal-Wallis $p$>0.99; $p$=0.40, respectively).

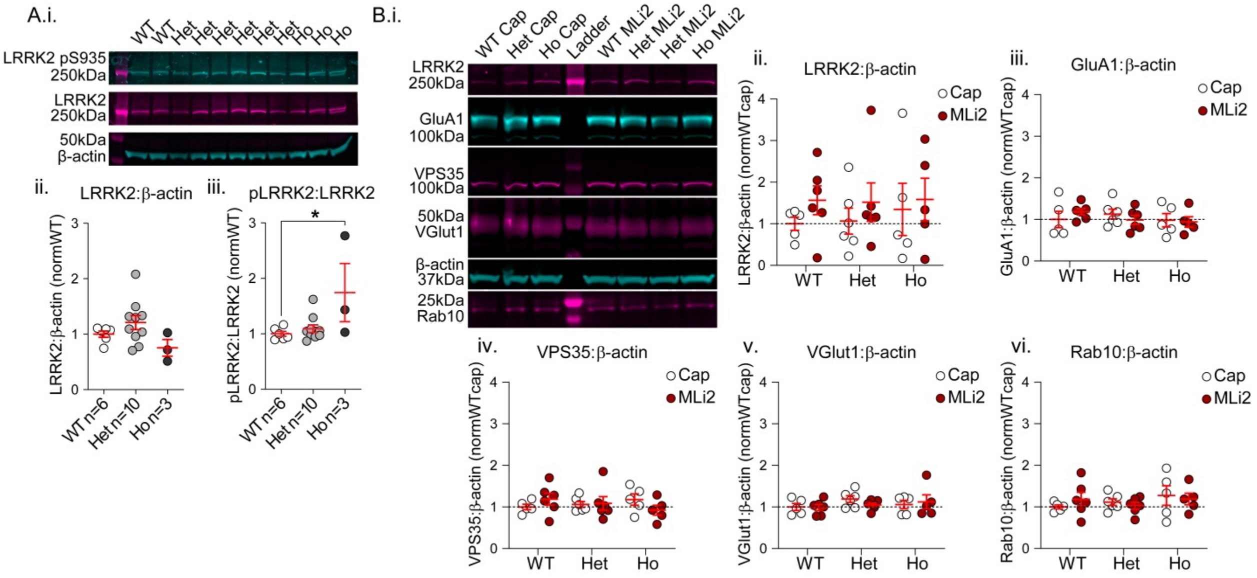


**Additional file 1: Figure S3 Phospho-LRRK2 is increased in VKI and MLi-2 does not alter protein levels.**

**A)** Western blot of LRRK2 pS935, LRRK2, and β-actin (i) revealed no genotype effect on LRRK2 expression levels (ii, Kruskal-Wallis $p=0.09$). LRRK2 pS935 levels were significantly altered, due to an increase in phosphorylation in homozygous tissue (iii, 1-way ANOVA $p<0.03$; Uncorrected Fisher's LSD $*p<0.02$). **B.i)** Whole brain lysates from VKI animals after acute LRRK2 kinase inhibition were blotted for LRRK2, GluA1, VPS35, VGluT1, Rab10, and β-actin (loading control). There were no significant effects of genotype or treatment on levels of LRRK2 (ii, 2-way ANOVA genotype x treatment $p=0.93$; genotype $p=0.90$; treatment $p=0.24$), GluA1 (iii, 2-way ANOVA interaction $p=0.45$; genotype $p=0.61$; treatment $p>0.99$), VPS35 (vi, 2-way ANOVA interaction $p=0.23$; genotype $p=0.94$; treatment $p=0.89$), VGluT1 (vii, 2-way ANOVA interaction $p=0.55$; genotype $p=0.39$; treatment $p=0.69$), or Rab10 (iv, 2-way ANOVA genotype x treatment $p=0.5258$; genotype $p=0.4683$; treatment $p=0.9659$). For Bii-vi, WTCap n=5, WTMLi2 n=6, HetCap n=6, HetMLi2 n=6, HoCap n=5, HoMLi2 n=5.

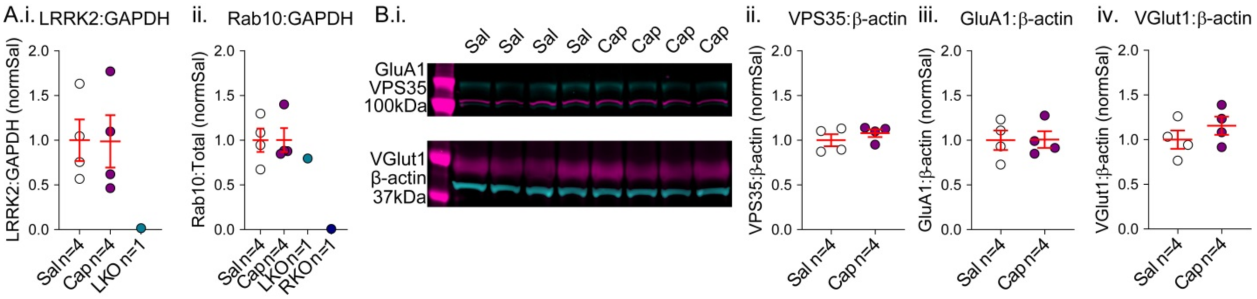


**Additional file 1: Figure S4 Captisol does not affect protein levels. A)** Further analysis of blots from Figure 1. There were no significant genotype effects on the level of LRRK2 (i; Mann-Whitney $p>0.99$) or Rab10 (ii; Mann-Whitney $p=0.89$). **B)** Western blots of WT brain lysate following acute treatment with Captisol or saline, probed for GluA1, VPS35, VGluT1, and β-actin (loading control). There were no significant effects of Captisol treatment on protein levels of VPS35, GluA1, or VGluT1 (ii-iv; Mann-Whitney $p=0.49$; $p>0.99$; $p=0.49$, respectively).

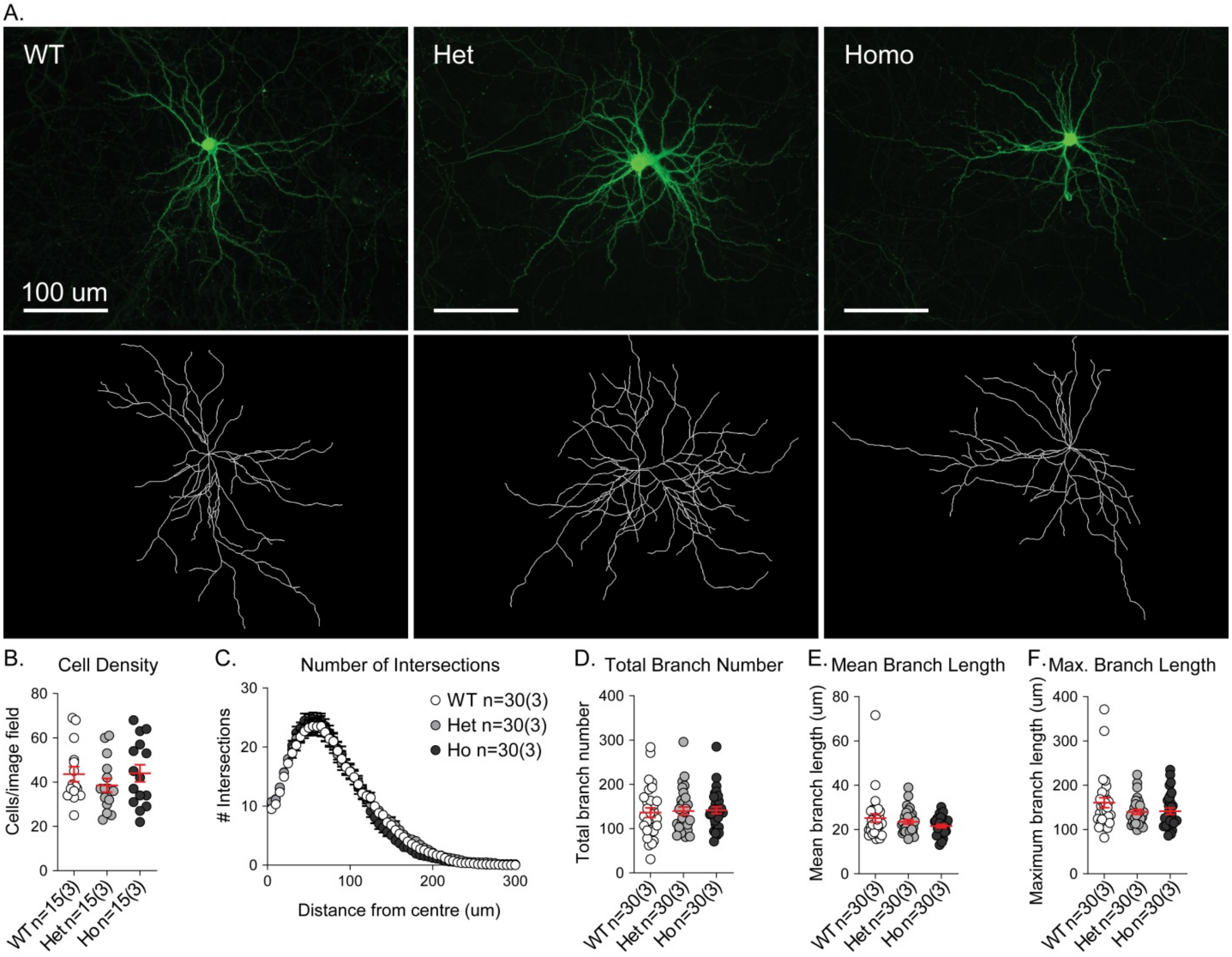


**Additional file 1: Figure S5 Cell density and dendritic morphology are not altered in VKI. A)** Cortical cells were nucleofected with CAG-AAV-GFP plasmids on the day of plating and fixed at DIV21. GFP signal was amplified and imaged (top panel), then 2D *in silico* cell reconstruction performed in ImageJ (bottom panel). **B)** There was no effect of genotype on neuron density, indicating equivalent survival and no cell death (1-way ANOVA $p$=0.47). **C)** Sholl analysis revealed no significant effect of genotype upon neurite complexity (2-way RM ANOVA radial distance x genotype interaction $p$=0.87; genotype $p$=0.78). **D-F)** There were also no genotype effects on total branch number, average branch length, or maximum branch length (Kruskal-Wallis $p$= 0.79, 0.34 & 0.29, respectively).

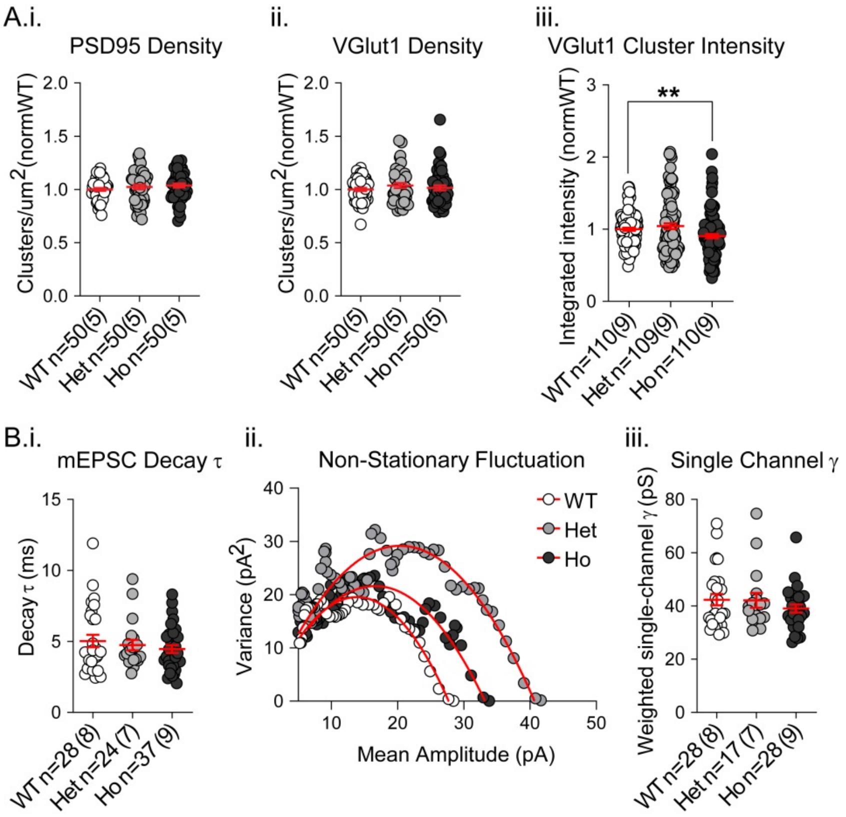


**Additional file 1: Figure S6 VGluT1 cluster intensity reduced in VKI and channel kinetics not affected.**

**A)** Supplemental analysis from untreated cortical cell culture synapse staining presented in Figure 3. PSD95 and VGluT1 densities were not altered by genotype (i-ii, Welch's ANOVA $p$=0.29 & 0.42, respectively). There was a genotype effect on VGluT1 cluster intensity, due to significant reductions in homozygous cells only (iii, Kruskal-Wallis $p$<0.007; Uncorrected Dunn's **$p$<0.003). **B)** Supplemental analysis of whole-cell patch clamp recordings from cultured cortical cells presented in Figure 3. Mean mEPSC decay times (τ) were not affected by genotype (i, Kruskal-Wallis $p$=0.74). Peak-scaled non-stationary noise analysis was performed by plotting the mean variance of traces from the recording average amplitude (ii, representative mean-variance plots); the best fit curve allows for the calculation of weighted single channel conductance of the synapses involved in each recording. Calculation of weighted single channel conductance from best-fit curves revealed no genotype effect on single channel conductance (iii, Kruskal-Wallis $p$=0.78).

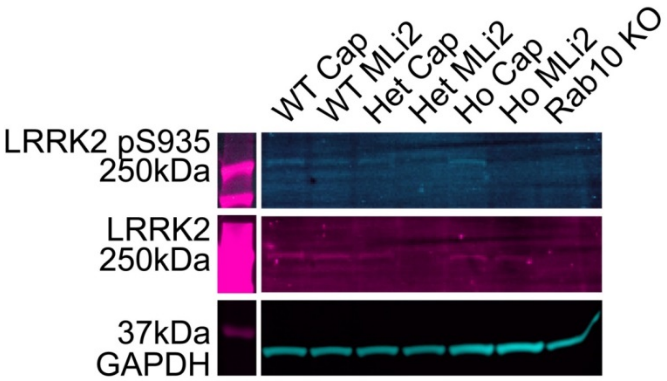


**Additional file 1: Figure S7 LRRK2 is expressed and phosphorylated in cultured neurons, and MLi-2 decreases pLRRK2.** Fluorscence western blot of pLRRK2, LRRK2, and GAPDH VKI cortical culture lysate revealed that the presence of LRRK2 and LRRK2 p935 in vehicle treated cultures, and absence of pLRRK2 following acute MLi-2 treatment; however, due to low stoichiometry bands were not high enough above background to be reliably quantified.

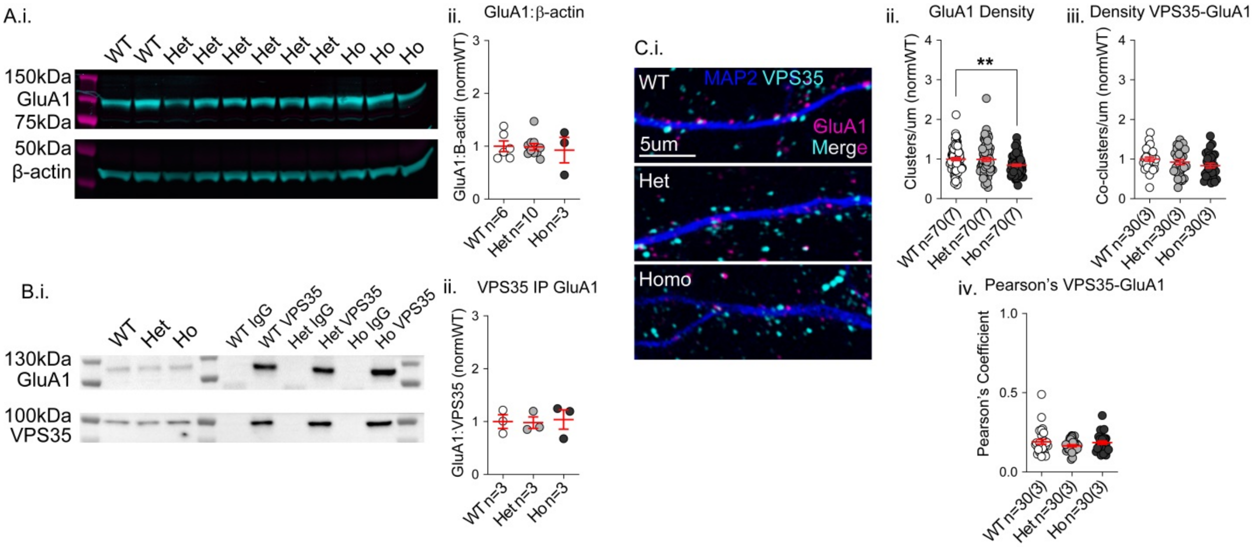


**Additional file 1: Figure S8 GluA1 levels unaltered but dendritic cluster density is reduced in VKI. A)** Western blot of GluA1 and β-actin in cortical lysates of VKI mice (i) revealed no genotype effect on GluA1 protein levels (ii, Kruskal-Wallis $p$=0.99). **B)** Co-immunoprecipitation of GluA1 with VPS35 (i) revealed no genotype effect (ii, Kruskal-Wallis $p$=0.99). **C)** Cultured cortical neurons immunostained for MAP2 (blue), VPS35 (cyan), and GluA1 (magenta)(i). There was a significant reduction in GluA1 cluster density in homozygous VKI neurons (ii, Kruskal-Wallis $p$<0.02; Uncorrected Dunn's **$p$<0.005) and no genotype effect on VPS35-GluA1 co-cluster density of (iii, 1-way ANOVA $p$=0.13), or Pearson's coefficient (iv, Kruskal-Wallis $p$=0.57).

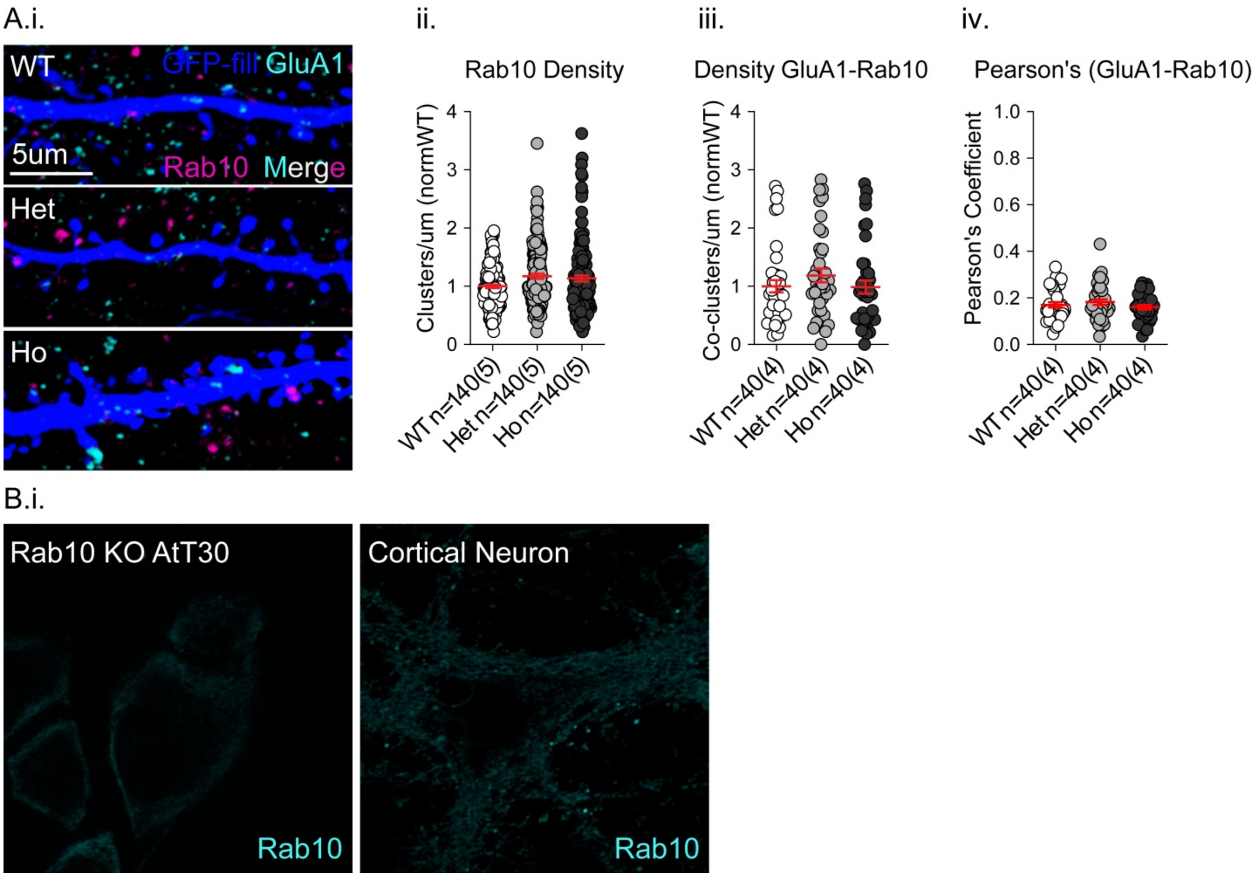


**Additional file 1: Figure S9 Rab10 does not colocalize strongly with GluA1 in cortical neurites. A)** GFP-filled (blue) cortical neurons immunostained for Rab10 (magenta), and GluA1 (cyan)(i). Rab10 cluster density was increased in both mutant genotypes, falling just shy of statistical significance (ii, Kruskal-Wallis $p<0.06$). There was no effect of genotype on co-cluster density or Pearson's coefficient (iii-iv, 1-way ANOVA $p=0.24$; Kruskal-Wallis $p=0.56$, respectively). **B)** Knock-out testing of specificity of Rab10 antibody for immunocytochemistry. Cultured cortical neurons and Rab10 knock-out AtT30 cells were stained by immunocytochemistry for Rab10, demonstrating punctate staining in the cortical neuron that is absent from the knock-out cells.
