# Supplementary material for: Endosomal traffic and glutamate synapse activity are increased in VPS35 D620N mutant knock-in mouse neurons, and resistant to LRRK2 kinase inhibition
Source: Mol Brain. 2021 Sep 16;14:143. doi: 10.1186/s13041-021-00848-w (PMC8447518; doi:10.1186/s13041-021-00848-w)

Western blots from Figure 1

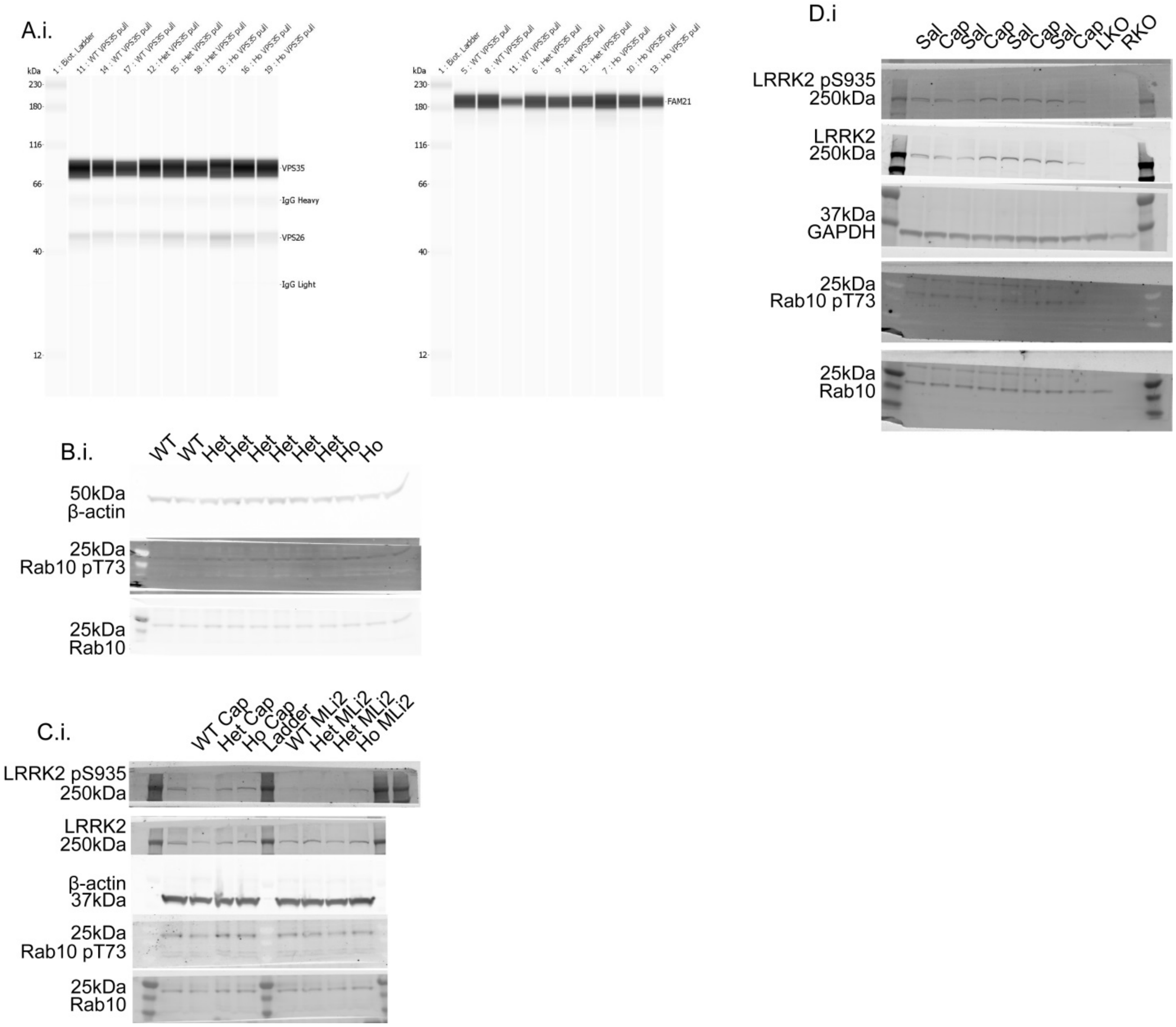

Western blots from Figure 3

C.i.

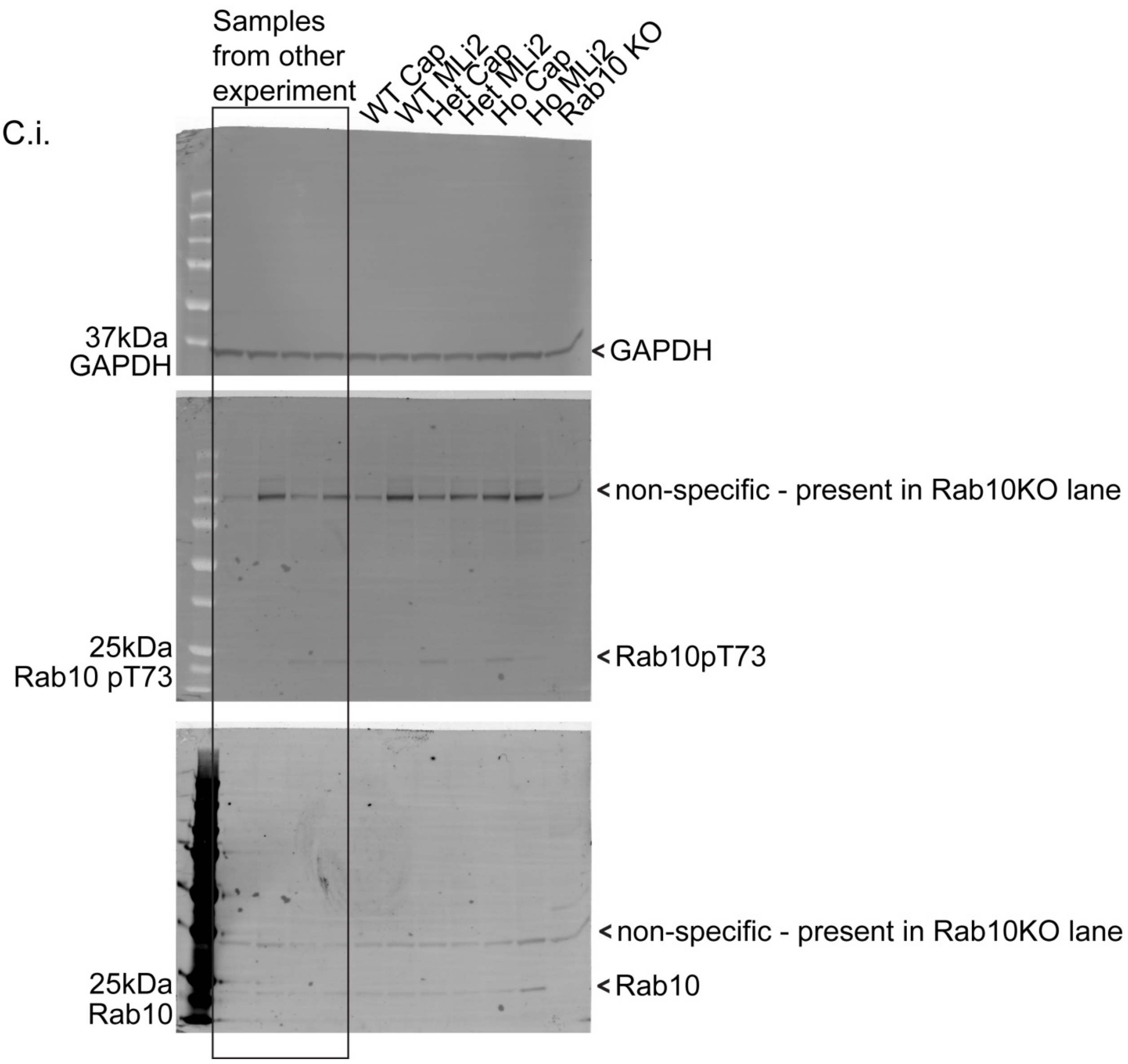

Western blots from Supplemental Figure 1

A.

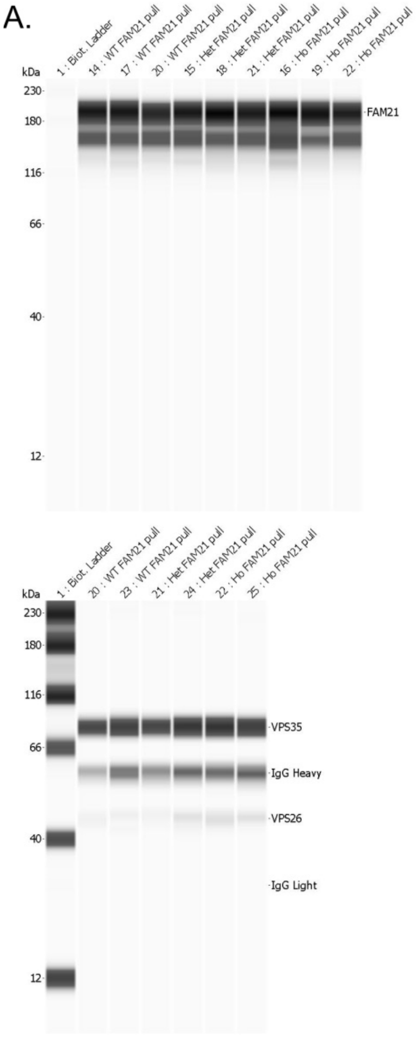

B.i.

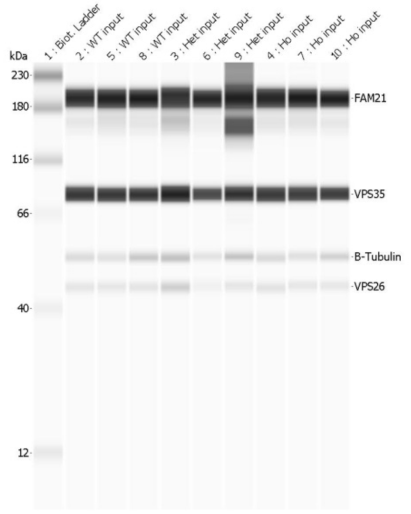

Western blots from Supplemental Figure 2

A.i.

STR

INPUTS

LRRK2

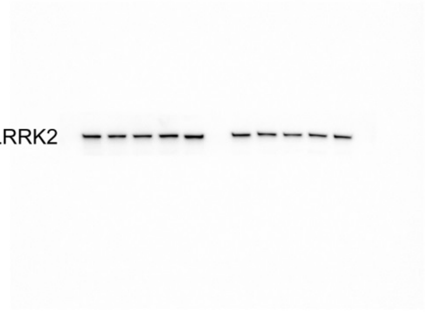

VPS35

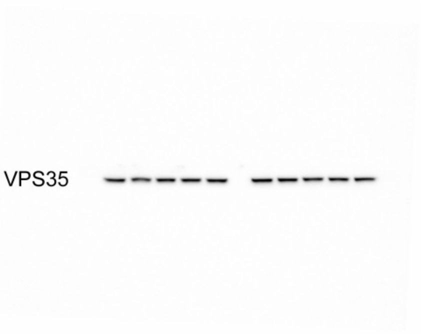

D2R

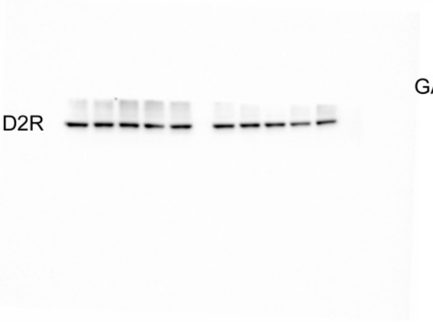

GAPDH

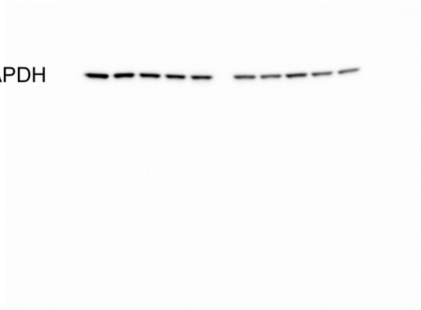

IPs

LRRK2

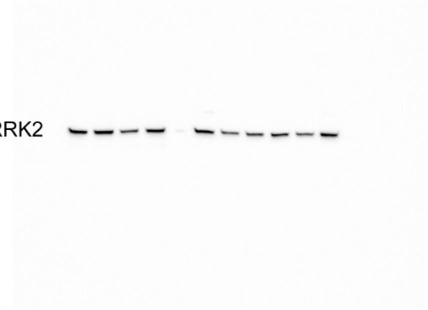

VPS35

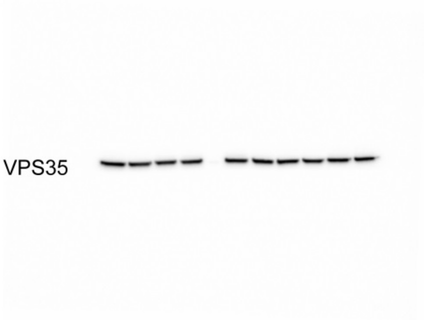

D2R

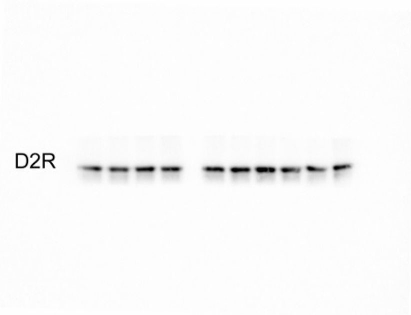

GluN1

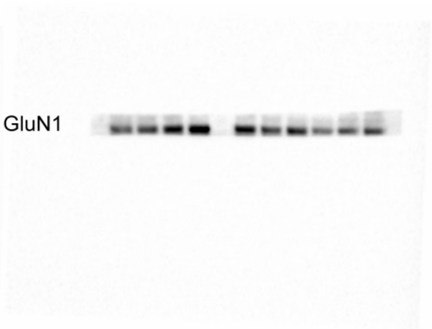

Combined

GluN1 exp for input

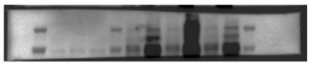

GluN1 exp for pull

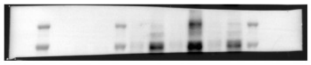

GluA1

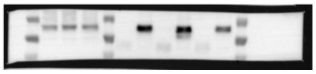

Western blots from Supplemental Figure 3

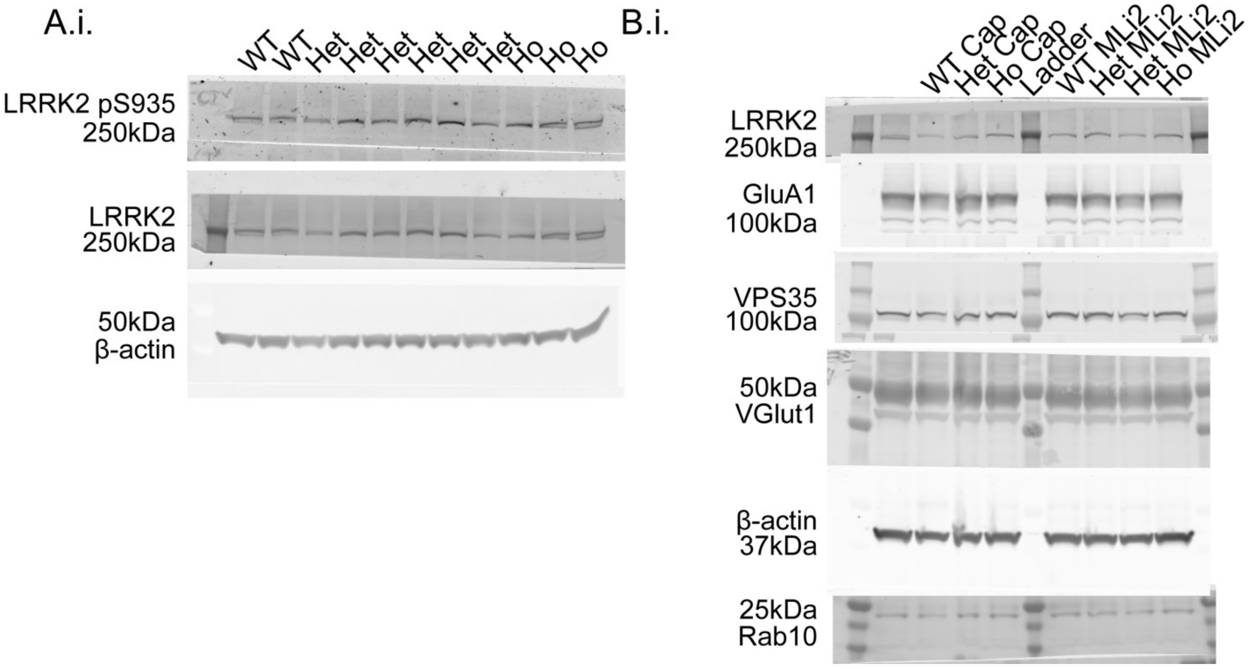

Western blots from Supplemental Figure 4

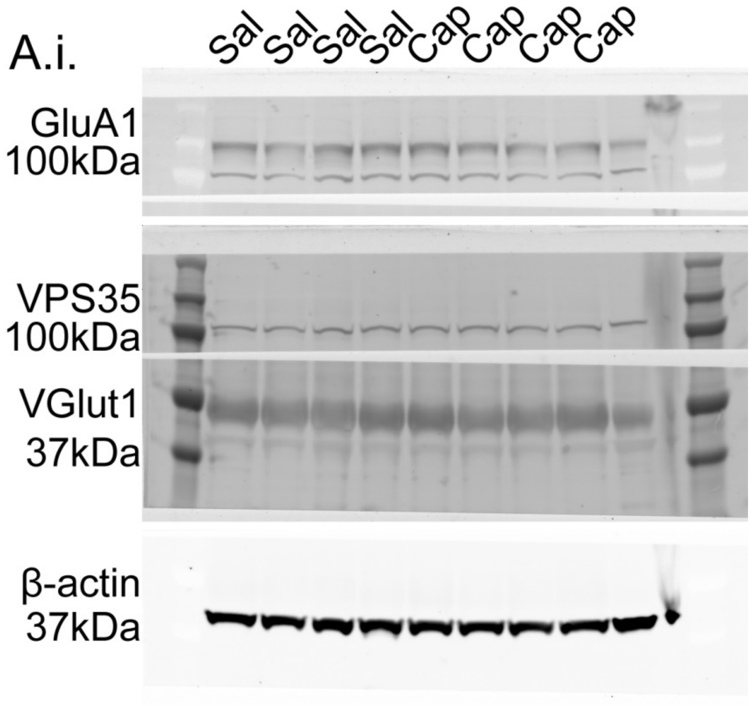

Western blots from Supplemental Figure 7

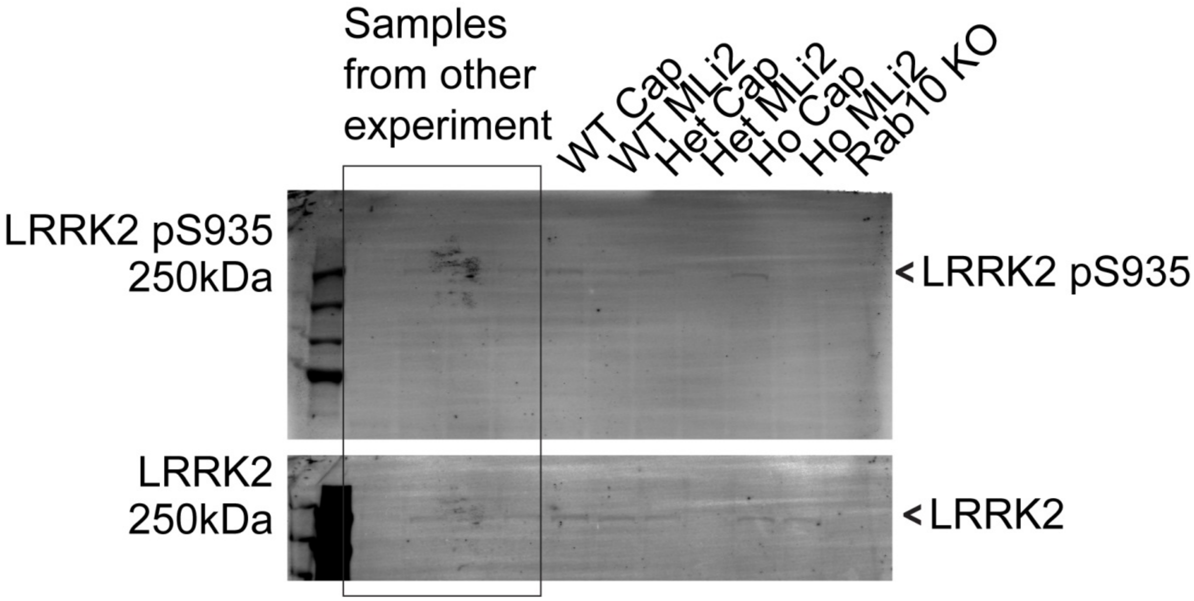

Western blots from Supplemental Figure 8

A.i.

150kDa
GluA1

50kDa
β-actin

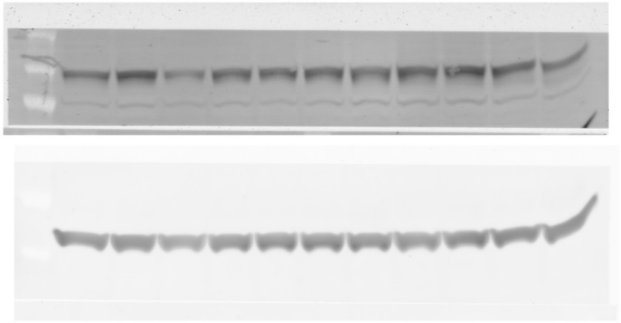

B.i.

130kDa
GluA1

100kDa
VPS35

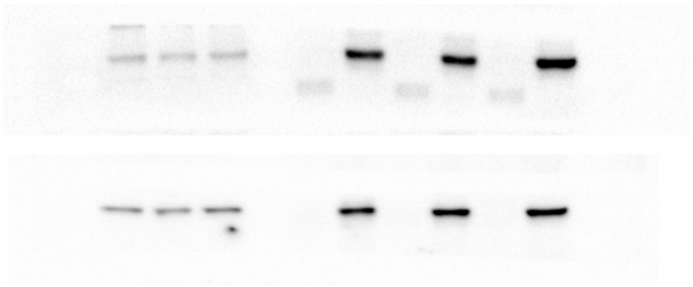

Supplement: Supplementary file 2 — Additional file 2: Western blot images. Original western blot images. [file 13041_2021_848_MOESM2_ESM.pdf]
